# CNS Repopulation by Hematopoietic-Derived Microglia-Like Cells Corrects Progranulin deficiency

**DOI:** 10.21203/rs.3.rs-3263412/v1

**Published:** 2023-09-12

**Authors:** Pasqualina Colella, Ruhi Sayana, Maria Valentina Suarez-Nieto, Jolanda Sarno, Kwamina Nyame, Jian Xiong, Luisa Natalia Pimentel Vera, Jessica Arozqueta Basurto, Marco Corbo, Anay Limaye, Kara Lynn Davis, Monther Abu-Remaileh, Natalia Gomez-Ospina

**Affiliations:** 1 Department of Pediatrics, Stanford University School of Medicine, Stanford, CA, 94305; 2 Hematology, Oncology, Stem Cell Transplant, and Regenerative Medicine, Department of Pediatrics, Stanford University, Stanford, CA, 94305; 3 Department of Chemical Engineering, Stanford University, Stanford, CA 94305; 4 Department of Genetics, Stanford University, Stanford, CA 94305; 5 MedGenome, Inc, 348 Hatch Dr, Foster City, CA 94404; 6 The Institute for Chemistry, Engineering and Medicine for Human Health (Sarafan ChEM-H), Stanford University, Stanford, USA.

## Abstract

Hematopoietic stem cell transplantation can deliver therapeutic proteins to the CNS through donor-derived hematopoietic cells that become microglia-like cells. However, using standard conditioning approaches, hematopoietic stem cell transplantation is currently limited by low and slow engraftment of microglia-like cells. We report an efficient conditioning regimen based on Busulfan and a six-day course of microglia depletion using the colony-stimulating factor receptor 1 inhibitor PLX3397. Combining Busulfan-myeloablation and transient microglia depletion results in robust, rapid, and persistent microglia replacement by bone marrow-derived microglia-like cells throughout the CNS. Adding PLX3397 does not affect neurobehavior or has adverse effects on hematopoietic reconstitution. Through single-cell RNA sequencing and high-dimensional CyTOF mass cytometry, we show that microglia-like cells are a heterogeneous population and describe six distinct subpopulations. Though most bone-marrow-derived microglia-like cells can be classified as homeostatic microglia, their gene signature is a hybrid of homeostatic/embryonic microglia and border associated-macrophages. Busulfan-myeloablation and transient microglia depletion induce specific cytokines in the brain, ultimately combining myeloid proliferative and chemo-attractive signals that act locally to repopulate microglia from outside the niche. Importantly, this conditioning approach demonstrates therapeutic efficacy in a mouse model of GRN deficiency. Transplanting wild-type bone marrow into *Grn*^−/−^ mice conditioned with Busulfan plus PLX3397 results in high engraftment of microglia-like cells in the brain and retina, restoring GRN levels and normalizing lipid metabolism.

## Introduction

Hematopoietic stem cell transplantation (HSCT) is the recommended treatment for several genetic disorders with severe and rapid neurodegeneration, including several lysosomal storage disorders (LSDs) and peroxisomal disorders (PSDs)^1,2^. HSCT is also a promising investigational therapy for other neurological diseases, including Friedreich’s ataxia^3^, Pelizaeus–Merzbacher disease^4^, and several neuronopathic LSDs^5^. Despite its proven benefit, HSCT’s use for neurological indications has been limited to a few fatal diseases because its risk-benefit assessment is usually unfavorable^1,6^. One of the caveats of HSCT is the common use of allogeneic cells (allo-HSCT) which exposes the recipient to immunological complications while often providing insufficient therapeutic correction^1^. To overcome this risk, autologous transplants of gene-modified HSPCs are being developed^7–9^. These approaches are demonstrating significant benefits in clinical studies leading to the approval of two autologous HSCT-based treatments for severe leukodystrophies (Metachromatic leukodystrophy and X-linked adrenoleukodystrophy)^7–9^. However, despite advancements in establishing sources of autologous cells, HSCT-based therapies still encounter obstacles related to their limited efficacy in the central nervous system (CNS) and delayed therapeutic onset.

How HSCT halts neurodegeneration is not completely understood, but the effect is partly due to bone marrow-derived cells that migrate to CNS, where they become long-lived resident myeloid cells, often called microglia-like cells (MGLCs)^10–13^. The mechanisms involved in the recruitment of hematopoietic-derived cells to the CNS are not yet entirely characterized, but myeloablative conditioning of the recipient is required for this process^10,14^. Myeloablation is commonly achieved in the clinic with Busulfan (BU), a CNS-penetrant DNA alkylating drug^7–9,15,16^. Even at the highest tolerated myeloablative dose, the combination of BU and HSCT, results in low, variable, and slow-paced engraftment of MGLCs in the CNS^17,18^. This modest and slow engraftment of MGLCs in the brain significantly limits the therapeutic efficacy of HSCT, further skewing the risk/benefit assessment unfavorably and preventing its broader applicability, particularly in diseases with neurodegenerative diseases with rapid neurological progression^19–22^. Accordingly, to improve the success of allogeneic and autologous HSCT for neurological indications, it is crucial to repopulate the CNS quickly and efficiently with MGLCs. This could enhance HSCT’s efficacy for the diseases for which it is currently used, and it may also offer a promising option for many other conditions that could theoretically benefit from an HSCT approach.

Several studies have examined the role of microglia depletion in overcoming the limited engraftment of bone marrow (BM)-derived MGLCs in the CNS^18,23,24^. The Colony-stimulating factor 1 receptor (CSF1R) is crucial for the survival of microglia (MG) and macrophages (MF) in rodents and humans^25–27^. Pivotal studies showed that the genetic depletion of CSF1R strongly favors the engraftment of BM-derived cells in the brain without any form of conditioning^23,28–30^. However, pharmacological depletion of MG and MF via inhibition of CSF1R (CSF1Ri) does not^23,31^. Several regimens that combine CSF1Ri with myeloablative total body irradiation or Busulfan have been reported, resulting in the near-complete replacement of microglia with BM-derived cells in mice^18,23,24,32–34^. Accordingly, combining myeloablation with CSF1Ri could represent a promising approach for pre-transplant conditioning in neurometabolic indications. However, available regimens need significant optimization for clinical use, and it is crucial to establish a regimen that includes the most appropriate reagents and dosage scheme for translational potential.

CSF1Ri is typically achieved by administering two main inhibitors through the chow, with prolonged courses lasting several weeks to months^18,23,24,33^. PLX5622 has been reported to affect hematopoiesis^35,36^, while PLX3397, approved by the FDA for the life-long treatment of individuals affected by tenosynovial giant cell tumor (TGCT) is predicted to have non-specific activity on other receptors^36,37^. Achieving efficacy with the fewest possible administrations is also crucial in optimizing a CSF1Ri regimen, as treatment-related toxicity depends on the dose and duration of treatment^36,37^. Moreover, considering the potential toxicities associated with CSF1Ri, a comprehensive evaluation of its impact on hematopoiesis and neurobehavior is essential to support its safety. Additionally, a better understanding of the kinetics of brain repopulation, the signaling molecules involved, and the characteristics of the cells repopulating the brain would provide insights to optimize microglia replacement by MGLCs and predict unwanted toxicities. Herein, we present our findings on an optimized and maximally effective conditioning regimen consisting of a short oral course of PLX3397, resulting in fast, robust, and long-term repopulation of the CNS by BM-derived MGLCs. This regimen does not negatively impact the hematopoiesis or neurobehavior in recipient mice. Our studies also determined the optimal timing of PLX3397 administration, the kinetics of MGLC repopulation, the signaling events in the brain in response to conditioning and repopulation, and the previously unrecognized heterogeneity of brain-engrafted MGLCs.

To assess the therapeutic potential of our conditioning regimen for a neurological disorder, we applied it to a mouse model of Progranulin (GRN) deficiency. In humans, insufficient GRN expression causes neurodegenerative diseases with an allele dose-dependent pattern^38^. Bi-allelic loss of function (lof) mutations cause Neuronal Ceroid Lipofuscinosis type 11 (CLN11, OMIM 614706), a rare LSD characterized by childhood-onset cognitive decline, retinitis pigmentosa, and early death^39^. Mono-allelic *GRN* lof mutations represent 5% of all cases of Frontotemporal dementia (GRN-FTD, OMIM 607485), an adult-onset disease presenting progressive changes in behavior, personality, and language, ultimately leading to early death^40–44^. CLN11/FTD represents a critical unmet need and is potentially amenable to protein, gene, and cell therapy approaches. However, the effectiveness of these approaches is challenged by the difficulty in delivering the large GRN protein across the blood-brain barrier (BBB), achieving widespread GRN distribution in the CNS^45–48^, and by potential complications of supraphysiological or ectopic GRN expression^47,49^.

GRN is a highly secreted lysosomal protein that is constitutively expressed but primarily enriched in microglia in the CNS^50,51^. Importantly, secreted GRN can be taken up by neighboring cells via receptor-mediated endocytosis^52,53^. Accordingly, HSCT-based microglia replacement could be an efficacious treatment for GRN deficiency, as secreted GRN from MGLCs can cross-correct other cells while concomitantly replacing diseased microglia^54–57^. Furthermore, by maintaining the expression within a cell type that typically expresses high levels of the protein, an HSCT-based approach could be safer as it minimizes potential complications of ectopic expression. Herein, we provide unprecedented results that transplantation of wild-type cells restores therapeutic GRN levels in the CNS of *Grn*^−/−^ mice and corrects defects of brain lipid metabolism when combined with an optimized, clinically translatable conditioning regimen based on Busulfan and PLX3337.

### Robust Engraftment of Bone Marrow-Derived Microglia-Like Cells in the CNS with Busulfan Myeloablation and Short PLX3397 Treatment

Current protocols for replacing microglia differ in the type of CSF1R inhibitor used, its formulation, the time of initiation, which can range from 14 days to several months, and the duration of administration, which typically lasts 2 to 4 weeks^18,23,24,33^. To arrive at a protocol with the most translational potential, we first optimized these parameters. We focused on PLX3397 (Pexidartinib, PLX) as the CSF1R inhibitor since it is FDA-approved for treating TGCT and has demonstrated safety even with long-term administration^36,37^. We tested different PLX regimens in adult C57BL/6 mice transplanted with bone marrow (BM) from coisogenic mice expressing green fluorescent protein (CAG-GFP). Consistent with previous reports, CSF1Ri alone did not result in donor cell engraftment in the brain or bone marrow following either intravenous bone marrow transplant (BMT) or intracerebroventricular delivery (ICV) of lineage-negative BM cells^18,23,31^ (**Extended data Fig. 1a-b**). When combined with myeloablation using Busulfan (BU), flow cytometry analysis of freshly isolated microglia showed that a short, 6-day course of 100 mg/kg/day PLX administered 15 days post-transplantation via gavage achieved the highest proportion of GFP+ CD45+CD11b+ MGLCs in the brain compared with several chow formulations and timing with respect to the transplant (**Extended data Fig. 1c-e, Supplementary Fig. 1a).**

**Figure 1.**
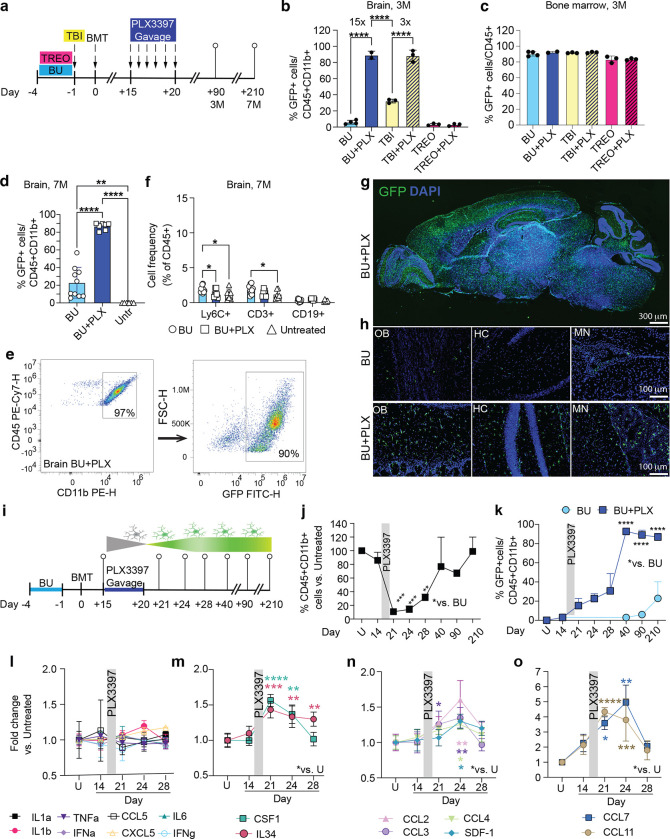
A short-course of PLX3397 enhances replacement of microglia by bone-marrow derived MGLCs and induces specific cytokines. **a,** Experimental timeline showing myeloablation of adult C57BL/6 mice with Total body irradiation (TBI, 10 Gy), Busulfan (BU, 100 mg/kg) or Treosulfan (TREO 5.5 g/kg). CSF1Ri was achieved with PLX3397 (PLX, 600 mg/kg, 100 mg/kg/day) by oral gavage at 15 days after total bone marrow transplant (BMT). Adult homozygous C57BL/6-CAG-GFP mice were used as bone marrow (BM) donors. **b-c,** Donor chimerism measured by flow cytometry measured as the fraction of GFP+ cells out of total CD45+ CD11b+ cells in the brain (**b**) or the fraction of GFP+ cells out of total CD45+ cells in the bone marrow (BM), 3 months (3M) post-BMT. **d,** Brain donor cell chimerism measured as the fraction of GFP+ out of total CD45+ CD11b+ cells and quantified at 7 months (7M) post-BMT by flow cytometry; Untr: Untreated mice. **e,** Representative flow plots, 7M post-BMT, of CD45+CD11b+ cells (left panel, gated on total CD45+ cells) and BMT-derived GFP+/CD45+ CD11b+ or MGLCs (right panel). **f,** Fraction of Ly6C+, CD3+ and CD19+ cells out of total CD45+ cells measured by flow cytometry in the brain 7M post-BMT and in untreated mice. **b-f**, each data point corresponds to one mouse. **g,** Representative sagittal section of the brain from one mouse treated with BU + PLX + BMT and analyzed 7M post-BMT. Green signal is the natural GFP fluorescence of transplant-derived cells. **h,** Representative images of MGLCs repopulating the olfactory bulb (OB), hippocampus (HC), and meninges (MN), of mice conditioned with BU alone (top panels) or BU + PLX (bottom row) and analyzed 7M post-BMT. **i,** Experimental timeline showing BU myeloablation (100 mg/kg) of adult C57BL/6 mice and administration of PLX (600 mg/kg, 100 mg/kg/day) at 15 days after BMT. Adult homozygous C57BL/6-CAG-GFP mice were used as BM donors. **j,** Fraction of CD45+ CD11b+ cells/total CD45+ cells quantified by flow cytometry in the brain of mice treated with BU + PLX at the indicated time points. The quantification and statistics are relative to untreated mice (U). **k,** Fraction of GFP+ cells out of CD45+CD11b+ cells quantified by flow cytometry in the brain of mice treated with either BU or BU + PLX at the indicated time points. Untreated mice (U) were used as controls. **l-o,** Serial brain cytokine analysis performed by 48-plex Luminex assay or ELISA assay (IL34 and SDF-1), at the depicted time points (panel i). **l-o,** all cytokine quantifications and statistics are relative to untreated mice (U). **b-f, j-o**, Data are Mean ± SD. The number of mice is **a-b**, BU n=4, BU + PLX n=2, TBI n=3, TBI+PLX n=3, TREO n=3, TREO+PLX n=3; **d, f**: BU n=10, BU + PLX n=7, Untreated (Untr) n=10. **i-o**, n=3 per time point. Statistical analysis: **b, d, f,** one-way ANOVA with Tukey post-hoc; **c,** multiple t-test with Holm-Sidak correction**; j, l-o,** one-way ANOVA vs. Untreated with Dunnett post-hoc; **k,** two-way ANOVA with Sidak post-hoc.

To examine how this shorter PLX regimen performs under different myeloablation protocols, we compared CNS and hematopoietic engraftment in wild-type C57BL/6 mice conditioned with either total body irradiation (TBI, 10 Gy), BU (100 mg/kg), or Treosulfan (TREO, 5.5 g/kg), a non-CNS penetrant BU analog^10^ (Fig. 1a). Despite identical bone marrow chimerism, PLX administration significantly increased the fraction of MGLCs when combined with either BU (BU 6 ± 2.4% vs. BU + PLX 89 ± 4.9%, 15-fold increase) or TBI (TBI 32 ± 2.4% vs. TBI+PLX 88 ± 7%, 3-fold increase) resulting in near complete repopulation of the microglia niche (Fig. 1b–c). However, CSF1Ri did not increase CNS engraftment in mice conditioned with TREO (Fig. 1b, **Supplementary Fig. 1b)** supporting the idea that genotoxic damage of CNS-resident cells is needed for robust microglia replacement by hematopoietic-derived cells following CSF1Ri.

Our data indicated that using BU as the myeloablative agent, which is the standard drug for conditioning patients with neurometabolic disorders, along with a short course of PLX (BU + PLX), robustly increased the replacement of microglia with hematopoietic-derived MGLCs. To further improve and reduce the potential effects of CSF1Ri on hematopoietic reconstitution and intervention time, we tested the effectiveness of administering PLX before transplantation. Concomitant administration of PLX (day −6 to −1) with BU (day −4 to −1) resulted in a 5-fold increase in GFP+ cell chimerism in the brain compared to BU alone but was significantly less than when administered after transplant (15 ± 8% vs. 89 ± 5%, respectively, **Extended Data Fig. 1f-h).** Side-by-side comparison of PLX administration pre- and post-transplant in recipient *Cx3cr1*-GFP^+/−^ mice (expressing GFP in microglia and macrophages) transplanted with BM from CAG-RFP^+/−^ mice (ubiquitously expressing red fluorescent protein, RFP) demonstrated that post-transplant administration of PLX resulted in more efficient depletion of host GFP+ microglia and higher replacement by BM-derived RFP+ cells in the brain, despite similar BM chimerism (**Extended Data Fig. 1i-k)**.

Having confirmed that the best protocol combined BU-myeloablation with six days of post-transplant PLX administered via gavage, we focused on this regimen for subsequent analyses. We first examined the long-term persistence of cells by quantifying the engraftment of hematopoietic cells in the CNS and hematopoietic organs seven months post-BMT. At this time, there remained stable engraftment of GFP+ CD45+ CD11b+ MGLC cells in BU + PLX-treated mice which was significantly higher than in BU alone (87 ± 4% vs. 23 ± 17%, Fig. 1d–e). Notably, the microglia niche was replaced specifically by BM-derived myeloid cells (CD45+ CD11b+) as we did not find increased frequencies of Ly6C+ monocytes, CD19+ B cells, and CD3+ T cells in BU + PLX-treated compared to untreated mice (Fig. 1f). This suggests that brain repopulation by BM-derived CD45+ CD11b+ cells is a regulated process and not an indiscriminate infiltration of immune cells as observed in several neurodegenerative and autoimmune diseases^58,59^. Histological analysis showed a widespread and homogeneous distribution of BM-derived GFP+ cells throughout the brain, spinal cord, and retina in mice treated with BU + PLX (Fig 1g–h, **Extended data Fig. 2)**.

### Fast replacement of microglia by BM-derived MGLCs coincides with brain-specific cytokine induction.

A known limitation of HSCT for neurological indications is its delayed therapeutic effect^19–22^. Therefore, achieving fast repopulation of the CNS by hematopoietic cells would significantly improve HSCT’s efficacy for neurological diseases with rapid progression. To examine the kinetics of brain repopulation, we looked at freshly isolated microglia preparations at 1, 4, 8, and 20 days after PLX withdrawal (corresponding to 21-, 24-, 28-, and 40-days post-transplantation, Fig. 1i). Flow cytometry analyses showed a dramatically depleted microglia compartment followed by fast repopulation by GFP+ CD45+CD11b+ cells that peaked after 20 days (Day 40 post-transplant, Fig. 1j–k, **Supplementary Fig. 1c**). BU administration alone did not significantly reduce the number of microglia (86 ± 12%, D14 Fig. 1k) and the donor cell engraftment was substantially slower, reaching 2.9% 40 days post-transplant when the combination regimen achieved 93% (Fig. 1k). Microglia replacement by BM-derived cells in BU + PLX remained stable 7 months post-transplantation and was consistently higher than that achieved with BU alone (86.8 ± 4% vs. 23 ± 17%, Fig. 1k). Because BM is not always a clinical source of hematopoietic stem and progenitor cells (HSPCs) and the timing of repopulation might differ between BM and purified HSPCs, we also examined the kinetics of brain repopulation using Lin− KIT+SCA-1+ (LKS) HSPCs. The same time course analysis showed that fast microglia repopulation by MGLCs could also be achieved using primitive LKS HSPCs with similar kinetics as BM following myeloablation with either BU or TBI (**Extended data Fig. 3**).

Previous studies on cytokine secretion in the conditioned brain have focused on pro-inflammatory cytokines such as TNF-a IL1-b, IL1-a, and known myeloid chemokines such as CCL2 (also known as monocyte chemoattractant protein 1, MCP-1), CCL5, and CXCL10^11,17,60^. These studies consistently show that irradiation stimulates more pro-inflammatory cytokines than Busulfan. To elucidate mechanisms of recruitment and repopulation in our combined regimen, we measured a panel of 50 cytokines in the brain of BU + PLX-treated mice and compared them to untreated mice (U). Cytokine quantification at 1, 4, and 8 days after PLX3397 withdrawal showed unchanged levels of pro-inflammatory cytokines such as IL1-b, IL-1a, IL6, and TNF-a (Fig. 1l). One and four days after PLX, we found brain-specific and transient increases in CSF1R ligands CSF1 and IL34 (Fig. 1m), presumably reflecting the depletion of CSF1R-expressing cells and signals that promote niche repopulation. The chemokines CCL2, CCL3 (also known as macrophage inflammatory protein 1-alpha, MIP-1a), CCL4 (Mip-1b), CCL7 (MCP-3), and CCL11 were also transiently elevated, which is consistent with their role in promoting the mobilization of myeloid cells (Fig. 1n–o and **Extended data Fig. 4a**). Interestingly, SDF-1 (also known as CXCL12), a potent chemoattractant for hematopoietic cells^61^, was also increased the brain (Fig. 1n). Most cytokines, except IL34, BAFF, and CXCL10, returned to baseline by day 8 (Fig. 1m and **Extended data Fig. 4a**). The induction of CCL11, CCL7, and CXCL10 in the brain was not paralleled by increases in other pro-inflammatory cytokines normally co-induced during immune-derived inflammatory processes^62,63^ (Fig. 1l, o and **Extended data Fig. 4a**). Notably, the cytokine elevations following PLX withdrawal were brain-specific and were not detected in plasma (**Extended data Fig. 4b-f**). Overall, the pattern of cytokine induction differs from that observed in immune-derived inflammatory processes and suggests that myeloid proliferative and chemo attractive signals act locally to repopulate the depleted microglial niche.

### Hematopoietic Reconstitution and Neurobehavior following BU-myeloablation and Short PLX3397 Regimen

**Figure 2.**
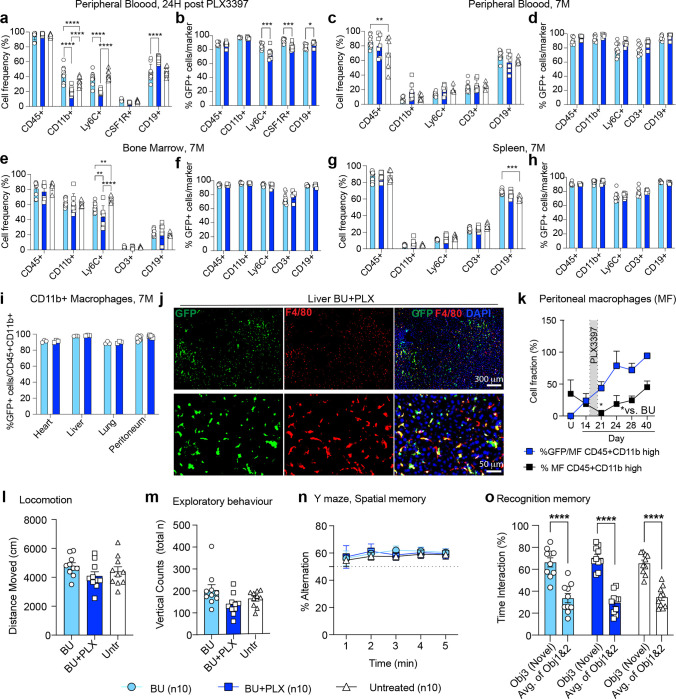
Normal hematopoietic reconstitution and neurobehavior following Busulfan myeloablation and a short PLX3397 regimen. **a-h**, Frequency of hematopoietic lineages and donor chimerism measured by flow cytometry in hematopoietic compartments and visceral organs of adult C57BL/6 mice untreated or transplanted with total bone marrow from C57BL/6-CAG-GFP mice and conditioned as depicted in Fig. 1a. Frequency and engraftment of myeloid cells (CD11b+, Ly6C+, CSF1R+), T cells (CD3+) and B cells (CD19+) was evaluated. The CNS chimerism of the same mice was described in Fig. 1. Analyses performed 24 hours after PLX withdrawal (corresponding to 21 days post-BMT) in peripheral blood (PB); BU (=10), BU + PLX (n=10); Untreated (n=10). **c-h,** Analyses performed 7M after BMT in peripheral blood (PB, **c-d**), bone marrow (**e-f**) and spleen (**g-h**); BU (n=10), BU + PLX (n=7); Untreated n=6. **i**, Donor macrophage chimerism in peripheral organs and peritoneum measured by flow cytometry 7M after BMT. The fraction of GFP+/CD45+CD11b+^high^ macrophages is depicted. Peritoneum: BU n=10, BU + PLX n=7, Untreated n=10; Heart, liver, and lung: n=3 mice/cohort. **j,** Representative images of GFP+ F4/80+ macrophages repopulating the liver of C57BL/6 mice treated by BU + PLX and analyzed 7M post-BMT. **k,** Depletion kinetics of host macrophages (GFP− CD45+ CD11b^high^) and engraftment of donor-derived macrophages (GFP+ CD45+ CD11b^high^) in the peritoneum of adult C57BL/6 mice before and after BU + PLX as measured by flow cytometry. The experimental timeline is depicted in Fig. 1i. n=3 mice/cohort; * vs. Untreated (U). **l-o,** Behavioral analyses performed between 6 and 7 months post-BMT in mice analyzed in Fig. 2 a–j; BU n=10, BU + PLX n=10, Untreated n=10. **l,** spontaneous locomotion analyzed using the Activity chamber test; the total distance moved (periphery plus center) is reported. **m,** exploratory behavior analyzed using the Activity chamber test; the total vertical counts (periphery +center) are reported. **n,** spatial memory analyzed using the three-arm Y-maze test; the percentage of spontaneous alternation across the arms is reported. **o,** recognition memory analyzed using the Novel object recognition test. The percent time spent interacting with the Novel object (Object 3) and the time spent interacting with previously encountered objects (Object 1 and 2) are reported. **a-i, k-o,** Data are reported as Mean ± SD. Statistical analysis: **a, c, e, g,** two-way ANOVA with Tukey post-hoc; **b, d, f, h, i,** multiple t-test with Holm-Sidak correction; **k,** one-way ANOVA vs. Untreated with Dunnett post-hoc; **l-o,** one-way ANOVA Tukey post-hoc.

The impact of CSF1Ri on hematopoiesis is currently a topic of debate^35,64,65^. Some studies have observed significant changes in the frequency of hematopoietic lineages over time^35^, while others have noted minor ones^66^. To better understand the impact of our optimized 6-day PLX3397 regimen on hematopoietic reconstitution after transplantation, we compared the frequencies of hematopoietic progeny using lineage-specific markers [CD11b (myeloid cells/macrophages), Ly6C (monocytes), CD3 (T cells), CD19 (B cells)] in mice that received BMT after conditioning with either BU or BU + PLX. As expected by the expression of CSF1R in CD11b+ and Ly6C+ subsets of myeloid cells^67–69^, 24 hours after PLX withdrawal, we observed an acute reduction in the respective fraction of these circulating myeloid cells with a compensatory increase in circulating B cells (Fig. 2a). Among these circulating cells most were GFP+ and there were small but statistically significant differences in the fraction of Ly6C+: 86 vs 74%, CSF1R+: 93% vs. 84% and, CD19+: 84 vs 89% between BU and BU-PLX conditioned mice respectively (Fig 2b). Seven months post-transplantation, the frequencies of hematopoietic lineages and the donor derived chimerism in hematopoietic peripheral blood (PB), bone marrow (BM) and spleen (SP), were the same between BU + PLX and BU mice, except for a 13% decrease in Ly6C+ cells in BM (Fig. 2c–h and **Supplementary Fig. 2a).** Time course analysis of hematopoietic reconstitution after transplant in BU + PLX conditioned mice showed full reconstitution of all lineages including CD41+ platelets and Ly6G+ granulocytes by day 40 post-transplantation (**Extended data Fig. 4g-h**).

We also examined the reconstitution of tissue macrophages in the heart, liver, lung, and peritoneum. Long-term engraftment of donor-derived GFP+ macrophages (MF) in these tissues had similar efficiencies in BU + PLX- and BU-treated mice (Fig. 2 i–j,). Kinetic analysis of the depletion and repopulation of peritoneal macrophages in BU + PLX-treated mice showed complete MF depletion at the time the drug was removed (day 21) and complete repopulation three weeks after (day 40, Fig. 2k and **Supplementary Fig. 2b**). Although no differences were found in the long-term chimerism in tissue macrophages, PLX accelerated macrophage replacement whether administered pre- or post-transplant (**Extended Data Fig. 4i-j**).

To further document potential toxicities of our regimen and the behavioral consequences of near-complete microglia replacement, we performed serial observations of appearance, activity, survival, and a battery of neurobehavioral tests. Consistently with TGCT individuals on PLX3397^36^, mice that received PLX3397 had well-demarcated patches of hair discoloration (**Extended data Fig. 4k**). We observed 100% long-term survival in all the mice receiving BMT and BU + PLX conditioning (n = 10 mice, seven months post-BMT, n= 5 mice, 12 months post-BMT). No differences were found between untreated, BU, and BU + PLX-conditioned and transplanted mice with serial assessments of spontaneous locomotion (activity chamber), exploratory behavior (Y-maze), and spatial and recognition memory (novel place-novel object recognition) (Fig 2. l–o). Overall, we found that BU-myeloablation combined with a short course of oral PLX3397 followed by transplantation does not significantly impact mouse motor and cognitive function.

### MGLCs are heterogeneous and express microglia-specific genes

**Figure 3.**
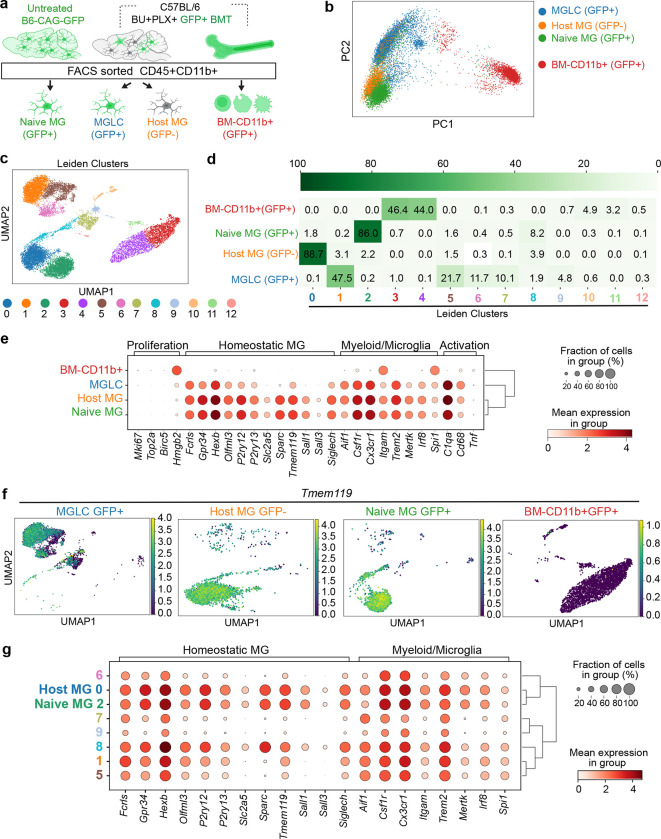
Single-cell transcriptional analyses of CD45+CD11b+ cells in the brain and bone marrow show the heterogeneity of MGLCs and the activation of microglia genes in the brain. **a,** Experimental design. Single-cell RNA sequencing (scRNA-seq) was performed on FACS-sorted CD45+CD11b+ cells isolated from mice that underwent BU + PLX conditioning and BMT as depicted in Fig. 1a. Four months after BMT, we isolated and compared four samples: 1) GFP+ CD45+ CD11b+ (**MGLC**), 2) GFP− CD45+ CD11b+ (host conditioned MG or **host-MG**), 3) GFP+ CD45+ CD11b+ from BM (**BM-CD11b+),** and 4) GFP+ CD45+ CD11b+ MG from untreated age-matched donor mice (**naïve MG**, Fig. 3a). **b,** Principal component (PC) analyses showing the clustering of MGLC, host-MG, naïve MG, and BM-CD11b+. **c, d** Leiden cluster analyses of MGLCs, host-MG, naïve MG, and BM-CD11b+ cells. **d**, Heatmap showing the cell fraction (Mean) contained in each Leiden cluster. The color of each cluster depicted in **c**, matches its number. **e,** Dot plot showing the differential gene expression of microglia signature genes in MGLCs, host-MG, naïve MG, and BM-CD11b+ cells. The dot size represents the percentage of cells expressing the gene in each sample and the color represents the gene average expression as depicted in the legend (right panel). **f,** UMAPs showing the expression of the microglia-specific *Tmem119* gene in MGLC (GFP+), host-MG (GFP−), naïve MG (GFP+) and BM-CD11b+ (GFP+) clusters. **g,** Dot plot showing the differential expression of microglia signature genes in the brain clusters depicted in panels **c-d**.

While transcriptional signatures that separate MGLCs and microglia have been described^18,70,71^, the single-cell heterogeneity of MGLCs engrafted in the brain following conditioning with BU/CSF1Ri has not been characterized. To examine this, we performed single-cell RNA sequencing (scRNA-seq) of FACS-sorted CD45+ CD11b+ cells isolated from mice that underwent BU + PLX and BMT and naïve mice. We compared four samples: 1) GFP+ CD45+ CD11b+ (**MGLC**), 2) GFP− CD45+ CD11b+ (host conditioned MG or **host MG**), 3) GFP+ CD45+ CD11b+ from BM (**BM-CD11b+),** and 4) GFP+ CD45+ CD11b+ MG from age-matched donor mice (**naïve MG**, Fig. 3a). To compare tissue- and ontogeny-specific signatures and heterogeneity, all four populations were first analyzed as a single dataset. Principal component analysis (PCA) showed that MGLCs are distinct from all the other CD11b+ populations but clustered closer to conditioned host MG and naïve MG than to BM-CD11b+ (Fig. 3b). Differential gene expression analyses showed that the CD11b+ cells within the four samples could be represented in 13 subpopulations (clusters) (Fig. 3c–d and **Extended Data Fig. 5a-c)**. The percentage of cells composing each cluster and cluster overlap is depicted in Fig. 3d. MGLCs separated into 6 main clusters [cluster 1 (47.5% of cells), 5 (21.7%), 6 (11.7%), 7 (10.1%), 8 (1.9%) and 9 (4.8%). Host MG was separated into 5 main clusters, with cluster 0 being the most abundant [cluster 0 (88.7% of cells), 1 (3.1%), 2 (2.2%), 5 (1.5%), 8 (3.9%). Naïve MG separated into one primary cluster [cluster 2 (86% of cells), and 3 less populated clusters [cluster 1 (1.8%), 5 (1.6%), 8 (8.2%). MGLC, host MG, and naïve MG showed shared cell subsets mainly in clusters 5 and 8 (Fig. 3d). BM-CD11b+ cells separated primarily into 4 clusters [cluster 3 (46.4% of cells), 4 (44%), 10 (4.9%) and 11 (3.2%), Fig. 3d] and did not significantly share cell subsets with the brain CD11b+ populations (Fig. 3d).

To understand and define microglia and MGLC transcriptional states, we examined the expression of signature genes reported across multiple studies (**Supplementary Table 1**)^28,72–78^. BM-derived cells that engrafted in the brain activated a transcriptional signature that was quite distinct from BM-CD11b+ (Fig. 3e). MGLCs expressed many classical homeostatic microglia genes like *Gpr34, Hexb, Olfml3, P2ry12/13, Siglech* and, *Tmem119* though expression levels were generally lower than in host and naïve MG (Fig. 3e–f). MGLCs also expressed other well-known microglia markers that are shared among cells in the myeloid/macrophage lineages like *Aif1, Csf1r, Trem2, Mertk and Cx3cr1* and had same levels of expression of activation genes (Fig. 3e). Ontogeny-specified microglia genes like *Sall1, Sall3,* and *Sparc* were expressed in embryonic-derived host and Naïve MG but were mainly absent in MGLCs (Fig. 3e), as previously reported^28,77^. Notably, we found that MGLCs expressed *Irf8* and *Spi1/Pu.1,* transcription factors known to specify microglia identity during embryonic development^77,79^. *Irf8* was expressed specifically in the brain (Fig. 3e), confirming its role as a master regulator of microglia and brain macrophages^77^. MGLCs did not express genes involved in cycling or proliferation, suggesting no expansion of this population at the time of the analysis (Fig. 3e). The gene expression signature that comprises the “microglia sensome”^73^ was also activated in MGLCs (**Extended data Fig. 5d).**

Analysis of microglia gene expression in the identified clusters revealed transcriptional heterogeneity within the MGLCs (Fig. 3g and **Extended Figure 5a-c**). The expression of several homeostatic microglia and myeloid/microglia genes was highest in MGLC clusters 1, 5, and 8 (Fig. 3g). Compared to host and naïve-MG, clusters 7 and 9 in MGLCs were the most divergent, expressing the lowest levels of *Irf8*, homeostatic microglia genes (e.g., *Siglech* and *Tmem119*), phagocytosis markers (*Mertk)*, and several myeloid/microglia markers (*Csf1r, Cx3cr1,* and *Itgam*, Fig. 3g). Overall, based on the expression of well-defined homeostatic microglia genes, the fraction of “homeostatic” cells within the MGLC population was estimated to be 83% which was lower than the 99% estimated for host MG (Fig. 3d–g). In addition to the combined analysis, we also performed differential gene expression analyses and evaluated the single-cell heterogeneity in each sample separately. Sequence processing and filtering of the four distinct datasets resulted in 40,734 individual scRNA profiles. This redemonstrated 12 distinct clusters in MGLCs and predicted 83% of homeostatic microglia (**Extended data Fig. 6a-g**). The relative abundance of each cluster (**Supplementary Table 2)** and the top 5 differentially expressed genes (DEGs) per cluster for each sample are shown (**Extended data Fig. 6b-e).**

### MGLCs upregulate genes characteristic of brain border-associated macrophages (BAM)

**Figure 4.**
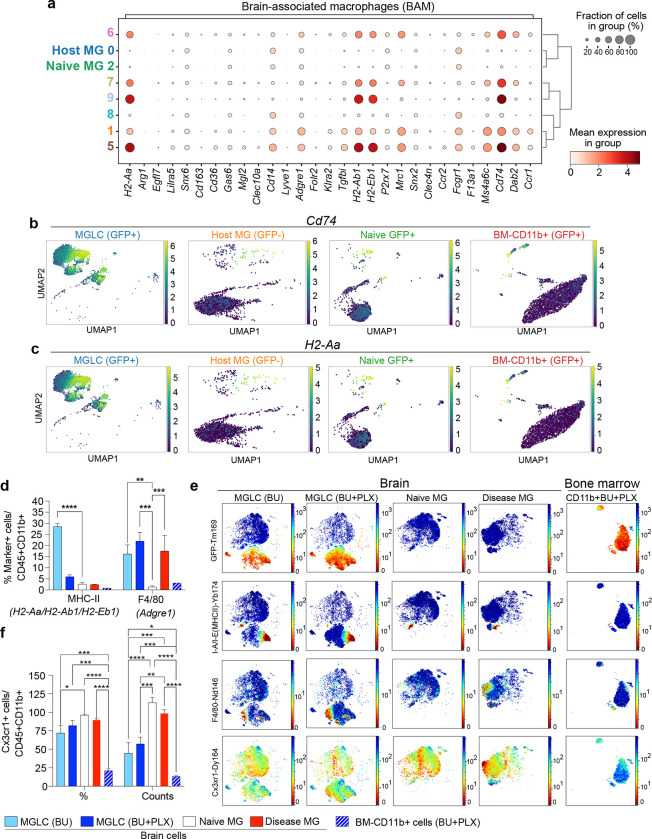
MGLCs upregulate genes characteristic of brain border-associated macrophages (BAM). **a,** Dot plot showing the expression of brain-associate macrophages (BAM) genes in the identified brain clusters. The dot size represents the percentage of cells expressing the gene in each sample and the color represents the gene average expression as depicted in the legend (right panel). **b-c,** UMAPs showing the expression of the *Cd74* (**b**) and *H2-Aa* (**c**) genes in MGLCs, host MG, naïve MG, and BM-CD11b+ clusters. **d-f**, Analyses of the expression of BAM and myeloid surface protein markers by high-dimensional CyTOF mass cytometry in CD45+ CD11b+ cells from brain and BM of transplanted mice 7M post-BMT. Groups compared: mice conditioned with either BU [MGLC (BU), n=2] or BU + PLX [MGLC (BU + PLX), n=2], untreated mice (naive MG, n=2), and age-matched mice with Mucopolysaccharidosis type 1, a neuropathic LSD with microgliosis (Disease MG, n=2). **d**, Percentage of CD45+CD11b+ cells positive for the BAM surface markers major histocompatibility complex (MHCII+) and F4/80 with the gene names in brackets. **e**, Optimized Stochastic Neighbor Embedding (Opt-SNE) plots showing the expression of the GFP (anti-GFP-Tm169 antibody), MHCII (anti-I-A/I-E Yb174 antibody), F4/80 (anti-F4/80 Nd146 antibody) and CX3CR1 (anti-CX3CR1-Dy164 antibody). **f**, Fraction of CD45+CD11b+ cells expressing CX3CR1 and Counts. **d and f,** Data are reported as Mean ± SD. Statistical analyses: one-way ANOVA with Tukey post-hoc.

The CD11b+ population in the brain also includes border-associated macrophages (BAMs) found in the perivascular space and leptomeninges^77,80^. Like microglia, BAMs derive from the yolk sac (except a subpopulation of choroidal plexus BAMs), rely on *Irf8* and *Spi1/Pu.1*, and are long-lived^7,70^. MGLC clusters 1, 5, 6, 7, and 9 were found to express many BAM genes, including MHCII genes (*H2-Aa/H2-Ab1/H2-Eb1), Tgfbi, Mrc1* (or *CD206*), *Ms4a6c, Cd74, Dab2, Adgre1 (or F4/80)* and *Ccr1.* However, many well-known BAM genes like *Gas6*, *Clec10a*, *Clec4n*, *Forl2, Lyve1*, and *Cd163* were not upregulated in MGLCs (Fig. 4a–b), suggesting a hybrid microglia/BAM transcriptional identity. MGLC clusters 5 and 9 showed the highest co-expression of MHCII (*H2-Aa/H2-Ab1/H2-Eb1)* and Cd74 (Fig. 4a–c), suggesting a microglia-like MHC^high^ phenotype. Cluster 6 and 7 may be classified as MHC^intermediate^, and cluster 1, the most abundant in MGLC, may be classified as MHC^low^; a finding consistent with the higher expression of several homeostatic MG genes this cluster. Except for *Tgfbi* and *Ccr1*, these BAM genes were not expressed in BM-CD11b+ cells, suggesting brain-specific upregulation (**Extended Fig. 7a).**

We next evaluated signature proteins expressed in BAMs, microglia, hematopoietic, and activated/proinflammatory immune cells in the brain and BM cells by high-dimensional CyTOF mass cytometry. The full antibody panel is described in **Supplementary Table 3.** To better understand the effect of CSF1Ri, we compared brain myeloid cells (CD45+ CD11b+) from mice treated with either BU alone, BU + PLX as well as naïve MG and disease MG from a neuropathic LSD characterized by microgliosis (Disease-MG)^81,82^. Intracellular cell staining with an anti-GFP antibody was used to distinguish host from BM-derived GFP+ cells. The engraftment of GFP+ MGLCs measured by CyTOF perfectly matched that measured by standard flow cytometry (88 ± 6% BU + PLX vs. 27 ± 9% BU alone), and analyses of immune cell markers did not show infiltration of proinflammatory cells in the brain of transplanted mice (**Extended Fig. 8).** Compared to naïve MG, the fraction of MHCII+ MGLC (GFP+) was highest in mice conditioned with BU alone (BU 29 ± 1.3% vs. vs. naïve 2.6 ± 0.7%, Fig. 4 d–e) and was slightly increased in BU + PLX - treated mice (6 ± 0.8%). The F4/80 surface marker (encoded by the BAM *Adgre1* gene) was expressed in a low fraction of naive MG (1.3%) but increased approximatively 15-fold in disease MG and MGLCs from both BU + PLX and BU-treated mice (Fig. 4d–e). Both cell surface markers were expressed in a small fraction of BM-CD11b+GFP+ cells (3.2%, Fig. 4 d–e). Interestingly, MHCII and F4/80 defined two distinct MGLC populations being rarely co-expressed (Fig. 4e, **middle panels**). The myeloid/microglia marker CX3CR1 was expressed on the surface of most MGLCs (72 ± 11% BU, 82 ± 7% BU + PLX), naïve MG (96 ± 0.8%), and disease MG (90±1.4% Fig. 4e–f), with MGLCs showing lower expression than naïve MG (Fig. 4f). A small fraction of BM-CD11b+ cells were positive for CX3CR1 (21 ± 2.5%, Fig. 4e–f) confirming the brain-specific induction of CX3CR1, as also demonstrated by sc-RNAseq (**Extended data Fig. 7b**).

### MGLCs upregulate genes characteristic of embryonic microglia

**Figure 5.**
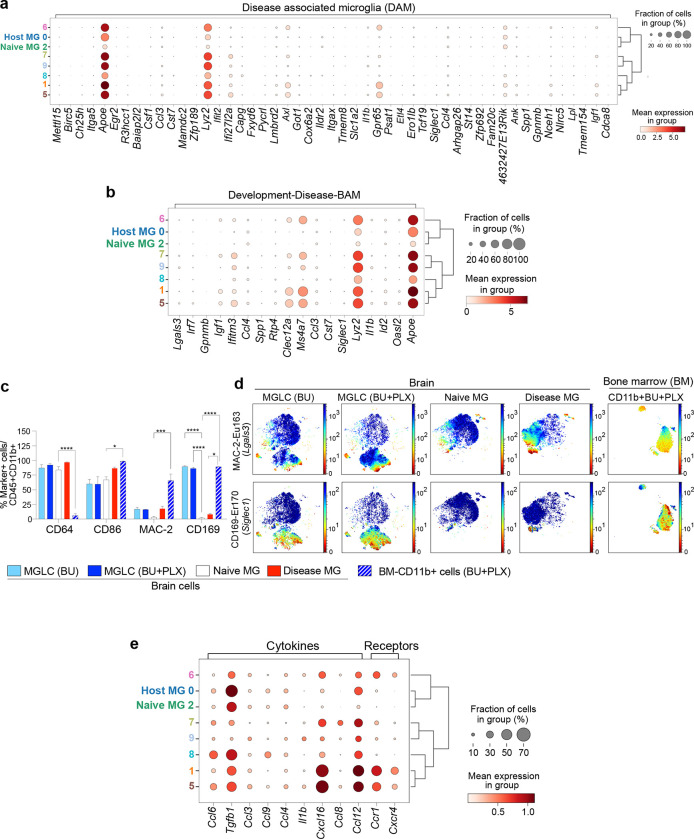
Genes upregulated in MGLCs and included in the disease-associated microglia (DAM) signature are characteristic of BAMs and embryonic microglia. **a,** Dot plot showing the expression of genes upregulated in the disease-associated microglia (DAM) signature (upregulation in DAM vs. microglia ≥3)^83^ in the identified brain clusters (depicted in Fig. 3 c–d). **b,** Dot plot showing the expression of genes upregulated by microglia during development, in BAMs and DAM in the identified brain clusters. The gene list for each signature are included in **Supplementary Table 1**. **c-d**, Analyses of the expression of brain-infiltrating monocyte-derived cells (MdCs) and brain-associate macrophages (BAMs) by high-dimensional CyTOF mass cytometry in CD45+ CD11b+ cells from brain and BM of transplanted mice 7M post-BMT. Groups compared: mice conditioned with either BU [MGLC (BU), n=2] or BU + PLX [MGLC (BU + PLX), n=2], untreated mice (naive MG, n=2), and age-matched mice with Mucopolysaccharidosis type 1, a neuropathic LSD with microgliosis (Disease MG, n=2). **c**, Percentage of CD45+CD11b+ positive for CD64, CD86, MAC-2, and CD169. **d**, Optimized Stochastic Neighbor Embedding (Opt-SNE) plots showing the expression of MAC-2 (anti-Mac2 Eu163 antibody) and CD169 (anti-CD169 Er170 antibody). **c,** Data are reported as Mean ± SD. Statistical analyses: one-way ANOVA with Tukey post-hoc vs. the GFP− WT untreated cohort (naïve MG). **e,** Dot plot showing the expression of selected genes encoding for cytokine and cytokine receptors that we found to be expressed in the identified brain clusters. The dot size represents the percentage of cells expressing the gene in each sample and the color represents the gene average expression as depicted in the legend (respective right panels).

A microglial transcriptional state defined as disease-associated microglia (DAM) has been identified in the brains of an Alzheimer’s Disease model^83^, and core genes of this signature have been reported to be upregulated in other brain diseases^84,85^. However, several genes in the DAM signature, such as *ApoE* and *Lyz2*, can be upregulated in microglia and BAMs in response to physiological stimuli, suggesting that some DAM genes may also reflect normal functions ^72,77,86^. We found a few DAM genes upregulated in MGLCs compared to naïve MG (Fig. 5a). *ApoE* and *Lyz2,* were highly expressed in most MGLC clusters and were also upregulated in conditioned host MG (Fig. 5a). Other DAM genes found to be increased in MGLC clusters were *Axl, Ifi27l2a, Gpr65* and *Igf1*. MGLCs did not express many other well-known DAM genes such as *Itgax* (CD11c), *Spp1* and *Clec7a*^83,84^ *(*Fig. 5a). Based on studies showing physiological upregulation of core DAM genes in embryonic microglia^72^, early postnatal microglia^72,86^, and in adult BAMs in healthy mice^77^, as well as studies reporting disease-associated signatures including disease-inflammatory macrophages (DIM)^87^ and old, proinflammatory microglia^72^, we curated a list of genes that we termed D-D-BAM (Development, Disease and BAM, **Supplementary Table 1**) and evaluated their co-expression in MGLCs (Fig. 5b). As compared to naïve MG, MGLCs consistently upregulated only genes that are physiologically induced in BAMs and embryonic microglia (*ApoE*, *Lyz2*, *Ms4a7*, *Clec12a*, *Ifitm3*, and *Igf1*^77^, Fig. 5b and **Extended Data Fig. 9a**). Therefore, the BM-derived MGLCs engrafted in the brain have a hybrid transcriptional identity co-expressing homeostatic adult microglia genes, BAM, and embryonic microglia genes.

Genes primarily expressed in monocyte and dendritic cells^77,86^ were not detected in the brain cell clusters, and no expression changes were found in MGLCs as compared to host and naïve MG (**Extended Fig. 9b and Supplementary Table 1**). High-dimensional CyTOF mass cytometry for markers upregulated in brain monocyte-derived cells (MdCs)^88^ like CD64^88^ and CD86^88^ showed no significant differences in GFP+ MGLC compared to naïve MG (Fig. 5c–d). We also examined CD169 (aka Siglec1) and MAC-2/LGALS3 (aka GALECTIN 3) as they are expressed in BAM and MdCs in the adult brain^88^ but not in healthy microglia^72,83–85,89^. The fraction of MAC-2+ CD45+ CD11b+ cells in the brain was the same in all conditions. Strikingly, CD169 stained most MGLCs and BM-CD11b+ cells but not naïve MG (Fig. 5c–d), therefore representing an MGLC-specific surface marker.

A comprehensive analysis of cytokine genes in the brain cell clusters showed increased expression of the anti-inflammatory *Cxcl16* cytokine gene^90^ in most MGLC clusters (1, 5, 7, 6) compared to both naïve and conditioned MG (Fig. 5e, and **Extended data Fig. 9c-d).** Interestingly *Ccl12* was only upregulated in conditioned host MG and MGLC but not in naïve MG (Fig. 5e and **Extended data Fig. 9c-d).** *Tgfb1* expression was reduced in most MGLC clusters while *Ccl6* was increased in a few MGLC clusters (5 and 8, Fig. 5e and **Extended data Fig. 9c-d)**. A combined analysis of cytokine and chemokine receptors revealed Ccr1 (cluster 1, 5, and 6, Fig. 4a) and *Cxcr4* as two additional receptors that may be used to distinguish some MGLCs from naïve MG upon BU + PLX conditioning (Fig 5f and **Extended data Fig. 9e-f**).

Taken together, MGLCs engrafted in the brain of BU + PLX-conditioned mice have a mixed transcriptional signature, expressing homeostatic microglia genes, BAMs, and embryonic microglia. MGLC subpopulations can be identified based on gene expression levels in gene sets. Furthermore, we have identified several surface markers of MGLC that can be used to differentiate them from host MG.

### Optimized BU + PLX corrects Progranulin deficiency and lipid metabolism in a mouse model of CLN11/FTD.

**Figure 6.**
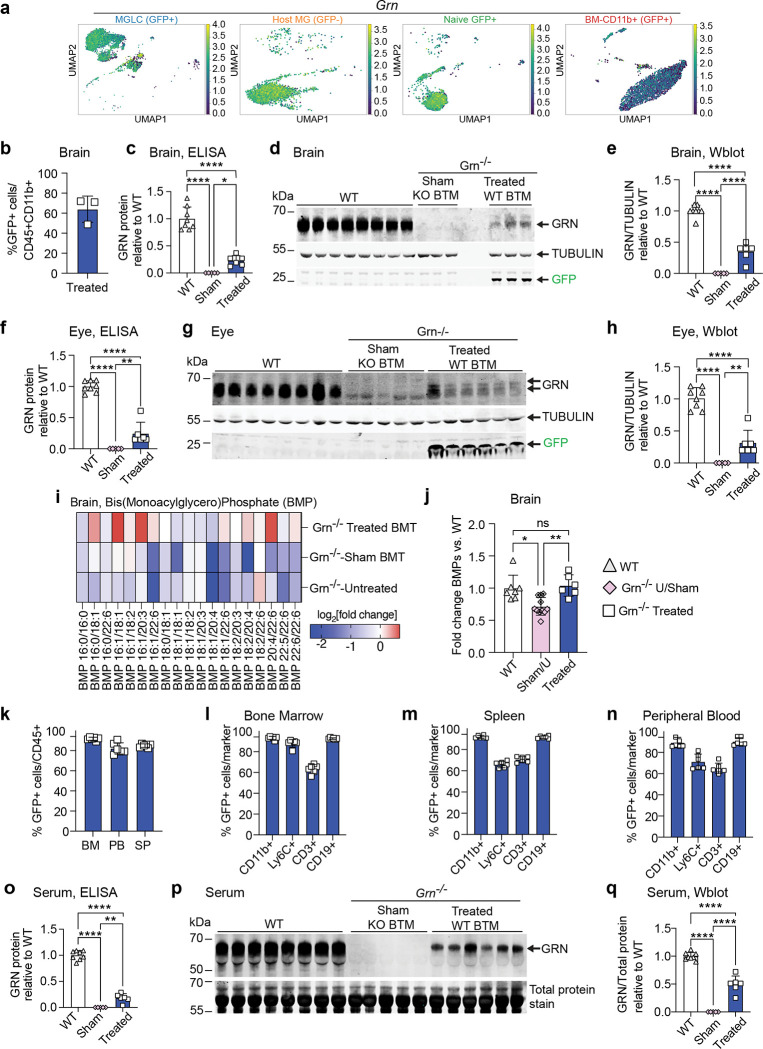
Correction of Progranulin deficiency and lipid metabolism in a mouse model of CLN11/FTD by wild type bone marrow transplant and optimized CNS conditioning. **a,** UMAPs showing GRN mRNA levels in MGLCs, host-MG, naïve MG, and bone marrow (BM)-CD11b+ (GFP+) clusters. **b-q,** Analyses of C57BL/6-*Grn*^−/−^ mice conditioned with BU (BU, 125 mg/kg, day −5 to day 0) and PLX3397 (PLX, 600 mg/kg, 100 mg/kg/day) via oral gavage 15 days after BMT. Comparison groups: 1) healthy controls were age-matched wild type C57BL/6 mice (WT, n=8), 2), disease mice were either left untreated (U, n=6) or conditioned with BU + PLX and transplanted with *Grn*^−/−^, KO BMT (Sham, n=5), and 3) Treated mice were *Grn*^−/−^ mice conditioned with BU + PLX and transplanted with WT BMT (Treated, n=6). **b,** brain donor MGLC chimerism depicted as the fraction of GFP+ out of total CD45+ CD11b+ cells and quantified 4 months post-BMT by flow cytometry. **c-e**, Quantification of mouse GRN protein by ELISA (**c**) and Western blot (**d-e**) in brain lysates of WT, Sham, and Treated mice. Quantification of GRN band intensities was normalized by alpha-Tubulin, the anti-GFP antibody reflects MGLC engraftment. **f-h.** Quantification of mouse GRN protein by ELISA (**f**) and Western blot (**g-h**) in eye lysates of WT, Sham, and Treated mice. Quantification of GRN band intensities was normalized by alpha-Tubulin, the anti-GFP antibody reflects MGLC engraftment. **i-j,** Targeted quantification of Bis(Monoacylglycero)Phosphate (BMP) in the frontal brain. BMP levels were normalized by total amount of POPC (1-palmitoyl-2-oleoyl-sn-glycero-3-phosphocholine). **i,** Heatmap showing the normalized amounts of BMP species relative to WT mice. **j,** Histogram showing the normalized amounts of total BMP species in control *Grn*^*−/−*^ mice (Untreated and Sham n=11) and Treated *Grn*^*−/−*^ mice (n=6). Dotted diamonds indicate sham-treated *Grn*^*−/−*^. Data are reported as fold change relative to WT mice. **k-n,** GFP+ donor chimerism in the hematopoietic compartments of Treated mice. **o-q**, Quantification of mouse GRN protein by ELISA (**o**) and Western blot (**p-q**) in the serum of WT, Sham, and Treated mice. **p-q**, Quantification of GRN band intensities was normalized by total protein. **b-j**, **o-q**, Data reported as fold change relative to WT mice. **b-q,** all data are Mean ± SD. Statistical analysis: one-way ANOVA with Tukey post-hoc (**c, e, f, h, k-o, q**); one-way ANOVA with Kruskal-Wallis post-hoc (**j**).

BMT/HSCT with a conditioning regimen that combines BU and CSF1Ri could be a promising treatment for Progranulin (GRN) deficiency. This is because microglia and MGLCs express and secrete high levels of GRN which can cross-correct other CNS cells (Fig. 6a). Moreover, the symptoms of severe deficiency (CLN11) affect the CNS and retina, where high replacement of dysfunctional GRN-deficient microglia can be achieved. However, a previous study in which mice were conditioned with TBI, BMT showed only partial efficacy, likely due to the low recruitment of hematopoietic cells to the brain^91^. To evaluate the effectiveness of our optimized BU + PLX conditioning, we transplanted a *Grn*^*−/−*^ mouse model^92^ with either wild-type BM from CAG-GFP mice (Treated, WT BMT) or BM from a *Grn*^*−/−*^ mice (Sham, KO BMT) mice. Four months after transplant, GFP+ MGLCs constituted up to 70% of CD45+CD11b+ cells in the brains of *Grn*^*−/−*^ mice and resulted in the reconstitution of 23–35% of wild-type GRN levels by ELISA and Western blot, respectively (Fig. 6b–e). Consistent with this, analyses of GRN expression in the eye also showed partial GRN restoration (24–32% of WT, Fig. 6f–h).

Loss of GRN results in decreased lysosomal Bis(Monoacylglycero)Phosphates (BMPs), regulatory lipids required for the activation of lysosomal hydrolytic enzymes involved in the catabolism of sphingolipids such as gangliosides^45,93^. This can lead to ganglioside accumulation in lysosomes, likely contributing to the neuroinflammation and neurodegeneration observed in CLN11 and FTD-GRN. To determine if BU + PLX + WT BMT could correct biochemical abnormalities observed in GRN deficiency, we measured BMPs in the cerebral cortex of transplanted mice. Mass-spectrometry analyses of brain BMPs confirmed a significant decrease in BMP levels in both Sham and Untreated *Grn*^*−/−*^ mice compared to WT (Fig. 6i–j). Most notably, BMP lipid metabolism was normalized in *Grn*^*−/−*^ mice receiving BU + PLX and WT BMT (Fig. 6i–j), suggesting that even partial reconstitution of GRN expression can restore lipid metabolism and result in therapeutic benefit.

Transplantation also resulted in biochemical correction in the periphery. The chimerism in hematopoietic organs was high (92% ± 1.6, 82% ± 5.6 and 85% ± 1.8 in BM, SP, and PB, respectively, Fig. 6k). Notably, the myeloid (CD11b+) and B-cell compartment (CD19+) were mostly donor-derived as seen in previous studies using wild-type recipient mice (Fig. 6l–n). However, compared to wild-type mice, T-cells and Ly6C+ had a higher fraction of host-derived cells (71% ± 2.7 in SP and 65% in PB, Fig. 6m–n). As seen in the brain and eyes, BU + PLX + WT BMT partially reconstituted circulating GRN to 18–49% of wild-type levels as measured by ELISA and Western blot (Fig. 6o–q).

To provide further evidence of phenotypic correction, we reviewed the literature to determine the deficits, timing, and magnitude of the behavioral phenotype in *Grn*^*−/−*^ mice^94–99^. Since the reported neurobehavioral phenotypes *of Grn*^*−/−*^ mice were variable, we performed serial neurobehavioral studies of *Grn*^*−/−*^ and wild-type mice every two months for 12 months. Analyses of spontaneous locomotion and exploratory behavior (Activity chamber), sociability (3-chamber sociability test), spatial memory (Y-maze spontaneous alternation), associative memory (passive avoidance test), cognitive deficit (nesting), and obsessive-compulsive behavior (grooming) showed no differences between wild-type and *Grn*^*−/−*^ mice (**Extended Data Fig. 10).**

In conclusion, our optimized BU + PLX achieves robust engraftment of MGLCs in the CNS, enabling wild-type cells to restore therapeutic levels of GRN in the brain and eyes and normalize lipid abnormalities in *Grn*^*−/−*^ mice.

## Discussion

Correcting protein deficiencies in the CNS remains a significant obstacle in treating neurological diseases due to the BBB. HSCT offers a promising solution by delivering therapeutic proteins to the CNS through donor-derived hematopoietic cells that naturally home as MGLCs after conditioning the CNS. MGLC engraftment has also been documented in humans who underwent myeloablative conditioning and allogenic HSCT for cancer treatment^13,70^ or MLD^100^. Interestingly, microglia replacement appears to be a normal physiological process that is commandeered after conditioning. Recent reports show that bone marrow-derived myeloid cells, resembling microglia, are protective in Alzheimer’s disease patients^101^. However, despite the therapeutic implications for a pathway that allows the controlled entry of myeloid cells into the brain, little is known about the mechanisms involved in the migration and differentiation of hematopoietic-derived MGLCs in the CNS.

The low, variable, and slow-paced engraftment of MGLCs in the CNS following standard myeloablation^17–22^ has been a key limitation of HSCT and has prompted the use CSF1Ri to enhance microglia-depleting agent and cell recruitment from the BM^18,23,24,32–34,102^. Here we report a highly efficient conditioning regimen based on BU and the shortest post-transplant course of CSF1Ri reported to date that results in reproducibly high (≥90%) and stable replacement of microglia by MGLCs throughout the CNS, including the neuroretina and the spinal cord. This protocol has, in principle, immediate clinical translatability. BU is the clinical agent for neurometabolic indications of HSCT, while PLX3397 is the only FDA-approved CS1R inhibitor with available safety data. The PLX3397 dose is well below what is prescribed for patients with TGCT (patients take ~1000 mg/day orally for 4 to 7 weeks^36^, while mice take ~3 mg/day for six days). Furthermore, since whole bone marrow is infrequently used as a cell source in the clinic and isolated CD34+ HSCPs are more prevalent, we also demonstrate that isolated, primitive HSPCs repopulate the CNS within a comparable timeframe as whole BM. This optimized conditioning protocol has the potential to improve the efficacy of allogenic HSCT and autologous transplantation of genetically corrected HSPCs and could expand HSCT’s application to more neurodegenerative disorders.

We also demonstrate that the timing of PLX3397 administration is key to maximizing MGLC chimerism. Maximum microglia replacement is achieved with PLX administered after full BU myeloablation and 15 days post-transplantation, suggesting that maximum replacement is achieved after fully “conditioning” the brain, a process that combines the myeloablative effect of BU and microglia depletion of CSF1Ri. The necessity for the pre-conditioning effect that BU has in the CNS is demonstrated by the lack of MGLC engraftment with a regimen that combines PLX3397 with non-CNS penetrant Treosulfan-based myeloablation^10^. How BU pre-conditions the CNS is poorly understood, but we and others showed that it does not cause overt BBB disruption^17,82^. At similar myeloablative doses, BU alters microglia morphology and induces the expression of senescence markers while reducing their regenerative capacity^33^. Consistent with this, the optimal timing of PLX3397 administration coincides with the peak of BU-induced microglia senescence reported by Sailor et al^33^. The data suggest that combining microglia depletion with a proliferative impairment of the microglia niche enables the competitive engraftment of BM-derived cells into the brain^23,33^. Nevertheless, we report that pre-transplant administration of PLX3397 was still better than BU alone and could benefit in situations where partial microglia replacement is sufficient to achieve a therapeutic effect.

Another limitation of HSCT with standard conditioning is its slow repopulation kinetics and delayed therapeutic onset in humans and mice^17–22^. This is particularly problematic for fast-progressing neurological diseases like infantile-onset Krabbe and other sphingolipidosis^19,20,22^. Using standard conditioning protocols, it takes 45 days for MGLCs to be detected in the mouse brain after transplantation, and it can take months for them to replace a small fraction of the microglia niche^18^. Our studies show that adding CSF1Ri leads to much faster microglia replacement by MGLCs. In mice treated with the combination regimen, repopulation surpassed BU alone on day 21 (17 days post-HSCT) and achieved peak repopulation (~90%) by day 40 (36 days post-HSCT), which remained stable after seven months. This rapid replacement of microglia allows for quicker delivery of therapeutic proteins and may prevent disease progression.

Post-transplant administration of PLX3397 at a 100 mg/kg/day dose for six days did not cause additional toxicities in mice conditioned with BU. These mice exhibited the same survival, spontaneous locomotion, exploratory behavior, and spatial and recognition memory as mice conditioned with BU alone. Despite reports on long-term effects of CSF1Ri on hematopoietic lineages (particularly for PLX5622)^35,65^, and the reported lack of receptor selectivity of PLX3397^36^, we did not observe significant adverse effects on hematopoietic reconstitution after a short course of PLX3397. Although PLX3397 inhibited KIT and FTL-3 receptors in vitro^103^, our data suggest a lack of functional impact in vivo in the transplantation setting. As both KIT and FLT-3 are required for hematopoietic stem cell (HSC) survival and differentiation, and anti-KIT therapies are being used to deplete HSCs^104,105^, the unaffected hematopoiesis and the lack of engraftment of donor cells in mice treated with PLX3397 alone suggest normal receptor function in HSCs. Ultimately, the inhibitor used, its receptor specificity, the total dose, and the treatment course may account for discrepancies in toxicity highlighting the need to limit dose and exposure time.

In addition to MG replacement, there is significant therapeutic potential for replacing tissue macrophages (MF) and using these cells to deliver therapeutic proteins to tissues such as the heart, lungs, and liver. In theory, a conditioning protocol that includes CSF1Ri could deplete tissue MF and improve the replacement of these cells after transplantation. By investigating the reconstitution of tissue MFs in various organs, we found that while the total engraftment of donor-derived MF was similar in mice treated with BU-PLX and BU alone, the addition of PLX3397 accelerated their repopulation. Interestingly, PLX3397 speeded MF replacement, whether administered post-transplant or concurrently with myeloablation suggesting recruitment mechanisms differ between the CNS and periphery and tissue MF replacement protocols could be further optimized for reduced toxicity.

While bulk transcriptome analyses have reported differences in gene expression between MGLCs and developmentally specified microglia^18,70,71^, the single-cell heterogeneity of MGLCs following conditioning with BU and PLX3397 had not been characterized. Consistent with previous studies, we found that BM-derived cells that engraft in the brain activated a transcriptional signature that was quite distinct from BM-CD11b+ cells and found no shared cell populations between cells in the BM and MGLCs, supporting the idea that environmental signals have a major role in dictating cell fate^28^. The MGLC population can be categorized into six main subpopulations based on differential gene expression. Notably, 83% of the MGLCs expressed homeostatic microglia gene signatures. We show that MGLCs acquire a hybrid homeostatic microglia/BAM/embryonic microglia signature. Most MGLC subpopulations express some genes commonly upregulated in BAMs. All MGLC populations showed increased expression of *ApoE* and *Lyz2*, which are increased in both BAMs and embryonic E14.5 microglia^72,77^. As observed in other studies, MGLCs do not express the ontogeny-specified transcriptional repressors *Sall1* and *Sall3*, which may contribute to the hybrid transcriptional state of MGLCs. However, there was a specific induction of the *Irf8*, a master transcriptional regulator of microglia and macrophages in the brain, which, together with *Pu.1*, also specifies the identity of microglia cells during embryonic development^77,79^. The hybrid transcriptional profile likely reflects differences in chromatin accessibility at these various loci established earlier during the development of microglia and the hemopoietic cells that repopulate in the CNS postnatally. Although the precise functional consequences of this hybrid phenotype need investigation, our behavioral data and the long-term outcomes of patients that have undergone transplantation suggest MGLCs can functionally replace microglia. Interestingly, recent studies propose a beneficial effect of bone-marrow-derived myeloid cells in the aging and AD brain^101,102,106^.

The signaling pathways involved in the migration, recruitment, and differentiation of hematopoietic-derived MGLCs in the CNS were unknown. Here we report several cytokines induced explicitly in the brain early after CSF1Ri. CSF1R ligands CSF1 and IL34 were transiently increased and may signal an “empty” niche to induce the proliferation of the endogenous repopulating pool. Several myeloid chemokines (CCL2, CCL3, CCL4, CCL7, and CCL11) were also transiently induced after PLX3397 withdrawal and may represent signals for the recruitment, expansion, or maturation of MGLC progenitors in the brain. Interestingly, a transient increase in SDF-1, a CXCR4 ligand known to mobilize HPSCs^61^, may also synergistically contribute to the overall CNS repopulation by BM-derived cells. We found no induction of cytokines observed in immune-derived inflammatory processes^62,63^. Together, the data suggests this is a highly regulated effort to repopulate and replenish the depleted microglial niche by combining myeloid proliferative and chemoattractive signals.

As the rapid and robust repopulation of the CNS by BM-derived cells could have important therapeutic implications, we also proved the therapeutic efficacy of the approach in a mouse model of GRN deficiency. In humans, GRN deficiency causes a spectrum of neurodegeneration ranging from childhood-onset (CLN11) to adult-onset disease (GRN-FTD), both of which lack effective treatments. Following BU + PLX, transplantation of a *Grn*^−/−^ model with wild-type bone marrow resulted in high engraftment of GFP+ MGLCs in the brain and retina, leading to partial reconstitution of GRN levels and restoring lipid metabolism in the brain. Furthermore, transplantation corrected biochemical abnormalities in the blood, suggesting correction in peripheral organs. Notably, MGLC engraftment in the CNS as well as T-cell and Ly6C+ cell chimerism were lower than observed in wild-type recipient mice. These could reflect disease-specific differences in response to conditioning, implying optimization might be required for every indication. These results provide crucial pre-clinical proof-of-concept for the efficacy of an HSCT-based strategy for CLN11/FTD-GRN.

## Methods

### Mice

Mice were housed in a 12-h dark/light cycle, temperature- and humidity-controlled environment with pressurized individually ventilated caging, sterile bedding, and unlimited access to sterile food and water in the animal barrier facility at Stanford University. All experiments were performed in accordance with National Institutes of Health institutional guidelines and were approved by the University Administrative Panel on Laboratory Animal Care (IACUC 33365). Experiments include male and female mice in roughly equal proportions.

### Conditioning

Adult C57BL/6J mice (Jax strain #000664) were conditioned with Busulfan (Sigma-Aldrich 14843), total body irradiation (TBI), or Treosulfan (MedChem Express HY-16503). Busulfan was administered intraperitoneally at a dose of 25 mg/kg/day for either 4 days (total dose 100 mg/kg) or 5 days (total dose 125 mg/kg) prior to transplant, as detailed in each study. Treosulfan was administered intraperitoneally at a dose of 1g/kg/day (500 mg/kg/dose, twice a day) for 5.5 days (total dose 5.5 g/kg) prior to transplant. Total body irradiation (TBI, 10 Gy, Kimtron IC-250) was performed 24 hours before transplant. PLX3397 (Pexidartib, MedChemExpress HY-16749) was administered via gavage (100 mg/kg/day) or PLX3397-complexed chow (290 ppm or 580 ppm in Envigo Teklad Diet 18%). The PLX3397-complexed chow was manufactured by Envigo Teklad. For oral gavage, the PLX3397 powder was resuspended in 100% DMSO and stored in aliquots at −80 °C; for administration the PLX3397 stock was diluted in a 1:1 mixture of Poly(ethylene glyco) Mn 400 (PEG400, Sigma-Aldrich 202398) and 1X Phosphate Buffered Saline pH 7.4 No Calcium/No Magnesium (abbreviated as PBS-1X).The specific formulation of PLX3397 and the treatment duration are detailed in the figure legends. The optimized PLX3397 conditioning regimen consisted in the administration of the drug 15 days post bone marrow transplant by oral gavage for 6 days (600 mg/kg, 100 mg/kg/day).

### Transplantation of total bone marrow

Bone marrow was harvested from adult C57BL/6-Tg(CAG-EGFP)131Osb/LeySopJ mice (Jax strain #006567) and transplanted in sex-matched adult C57BL/6J mice (Jax strain #000664). In one study total bone marrow was harvested from adult B6.Cg-Tg(CAG-mRFP1 mice (ubiquitously expressing RFP, Jax strain #005884) was transplanted in sex-matched adult heterozygous B6.129P2(Cg)-Cx3cr1tm1Litt/J mice (expressing GFP from the mouse *Cx3r1* locus, Jax strain #005582). Total bone marrow cells were isolated by flushing femurs and tibiae with PBS-1X (Thermo Fisher Scientific 10010049) supplemented with 4U/mL Heparin (H3149–500KU, Sigma Aldrich). After flushing the bone marrow cells were filtered using a 30 μm cell strainer, washed twice with PBS-1X and injected in total volume of 100 μL of PBS-1X (1.5×10^7^ cells/mouse). Mice were transplanted 24 hours after conditioning with Busulfan, Treosulfan, or TBI with intravenous injection of total bone marrow cells (1.5×10^7^ cells/mouse) in the retro-orbital sinus.

In one study eight-week-old B6(Cg)-Grntm1.1Aidi/J mice (Jax Strain #013175, abbreviated as Grn−/− or GRN KO, n=11) were conditioned with Busulfan (125 mg/kg, day −5 to 0,) and transplanted the day after with total bone marrow harvested from either sex-matched Grn−/− mice (Sham control mice n=5, 3 males and 2 females) or sex-matched adult C57BL/6-Tg(CAG-EGFP)131Osb/LeySopJ mice (Jax strain #006567). Grn−/− mice transplanted with wild-type GFP+ bone marrow (n=6, 3 males and 3 females) received PLX3397 by oral gavage 15 days after transplant (600 mg/kg, 100 kg/day). Untreated Grn−/− mice (n=6, 3 males and 2 females) and wild-type C57BL/6J mice (Jax strain #000664, n=10, 5 males and 5 females) were used as affected and unaffected controls.

### Systemic transplantation of Lin− Kit+ Sca1+ HSPCs

Primitive Lin− Kit+ Sca1+ HSPCs (LKS) were from adult C57BL/6-Tg(CAG-EGFP)131Osb/LeySopJ mice (Jax strain #006567) and cultured based on a published method^107^. Briefly, Kit+ cells were purified from total bone marrow (isolated as described above) using anti-Kit/CD117 microbeads and following manufacturer’s instructions (Miltenyi Biotec 130–097-146). Purified Kit+ cells were plated in cell bind plates (Costar 3337) at 5.5×10^5^ cells/mL and cultivated for 14 days in F12 media (Gibco 11765–054) supplemented with 100 ng/mL mouse TPO (Peprotech 315–14) 10 ng/mL mouse SCF (Peprotech 250–03), 0.1% Polyvinyl alcohol (PVA, Sigma-Aldrich P8136), 1% HEPES (Gibco 15630–080), 1% ITS-X (Gibco 51500–056) and 1% Penicillin-Streptomycin-Glutamine (Gibco 10378–016). LKS were maintained at 37 °C and 5% CO^2^ and half media changes were performed three times/week. LKS (5.5×10^5^ cells/mouse) were transplanted after 14 days in culture into sex-matched adult C57BL/6J mice (Jax strain#000664). Flow cytometry was used to evaluate the fraction of LKS in culture at day 3, 7 and 14 using the following antibodies: anti-mouse CD45 PE-Cy7 (clone 30F11 Thermo Fisher Scientific), anti-mouse SCA1/Ly-6A/E PE (clone D7 Thermo Fisher Scientific), anti-mouse KIT/CD117 APC (clone 2B8 Thermo Fisher Scientific), anti-mouse CD3 PB (clone 17A2, BioLegend), anti-mouse TER-119 PB (clone TER-119 BioLegend), anti-mouse Ly6C PacBlue (clone HK1.4 BioLegend), anti-mouse B220 PacBlue (clone RA3–6B2 Biolegend) and eFluor^™^ 780 (Thermo Fisher Scientific 65–0865-18) was used as viability dye.

### Intracerebroventricular transplantation of Lineage negative HSPCs

Lineage negative cells (Lin) were isolated from total bone marrow of adult C57BL/6-Tg(CAG-EGFP)131Osb/LeySopJ mice (Jax strain #006567) using the Mouse Lineage Cell Depletion Kit (130–090-858 Miltenyi Biotec) and transplanted in sex-matched adult C57BL/6J mice (Jax strain#000664). Immediately after the purification, Lin− cells were resuspended in PBS-1X at a concentration of 2×10^7^ cells/mL and kept on ice while injected in the mouse lateral ventricle using a stereotactic apparatus. A total of 5 μL/ventricle were injected (1 μL/min) to have a total dose of 1×10^5^ cells/ventricle.

### Flow cytometry analyses of donor chimerism

Mice were euthanized at various time points to analyze donor chimerism in tissues. Mice were anesthetized using a mixture of Ketamine/Xylazine (87.5 mg/kg Ketamine/12.5 mg/kg Xylazine, 0.1 mL/20 g intraperitoneal) and peripheral blood (PB) was collected using heparinized capillaries (Fisher Scientific, 22–260950) in PBS-1X supplemented with 10 mM EDTA pH 8.0 (Thermo Fisher Scientific 15–575-020), then, peritoneal cells were collected by washing the peritoneal cavity with 4 mL of PBS-1X. Femurs and tibiae, spleen, brain, liver, heart, lung and eye were collected after the transcardial perfusion of the anesthetized mice with PBS-1X. Erythrocytes in PB were precipitated using 2% Dextran (Spectrum Chemical D1004) in PBS-1X and the supernatant was collected in 5 mL of FACS-BL buffer [PBS-1X supplemented with 5% Fetal Bovine Serum (FBS, Thermo Fisher Scientific 16000069, and 1% BSA fraction V (Sigma-Aldrich 10735078001). Peripheral blood mononuclear cells (PBMCs) were obtained by centrifugation at 400g for 5 minutes. Bone marrow cells (BM, from femurs and tibiae) and splenocytes (SP) were extracted in RPMI (Thermo Fisher Scientific 61870127) supplemented with 10% FBS, 4U/mL Heparin (H3149500KU, Sigma Aldrich) and 0.2U/mL Dnase I (Worthington Biochemical Corporation LS002007) and filtered using a 30 μm cell strainer. Details of microglia and macrophage isolation are provided below.

Erythrocytes were lysed using the RBC lysis buffer (Thermo Fisher Scientific 00–4333-57). After RBC lysis the cells were washed and resuspended in MACS buffer and kept on ice for the following procedures. For flow cytometry staining, cells were resuspended in 10% vol/vol Mouse BD Fc Block^™^ (clone 2.4G2 BD Biosciences) 10 minutes and stained in the dark for 30 min using the following antibodies: anti-mouse CD45 PE-Cy7 (clone 104 Thermo Fisher Scientific), anti-mouse CD45 PE-Cy7 (clone 30F11 Thermo Fisher Scientific), anti-mouse/human CD11b-PE (clone M1/70 BioLegend), anti-mouse/human TER-119 PE-Cy5 (TER-119, BD Biosciences), anti-mouse Ly6C BV605 (clone AL-21 BD Biosciences), anti-mouse CD3 APC (clone 17A2 Thermo Fisher Scientific), anti-mouse CD19 PB (clone 6D3 BioLegend); anti-mouse Ly6G PE (clone 1A8 Thermo Fisher Scientific), anti-mouse CD41 PacBlue (clone MWReg30 BioLegend), anti-mouse CSF1R-APC (clone AFS98, Thermo Fisher Scientific); eFluor^™^780 (Thermo Fisher Scientific 65–0865-18) was used as a viability dye. After staining, cells were washed and resuspended in FACS-BL buffer. Stained cells were acquired using CytoFLEX (Beckman Coulter) and sorted using a MA900 Multi-Application Cell Sorter (Sony Biotechnology) flow cytometer. Flow cytometry data were analyzed using FlowJo software (FlowJo, LLC, USA).

### Microglia and macrophage isolation

Isolation of brain microglia was performed using a modified version of a reported method^108^. The brain was minced in ice-cold HBSS 1X (Thermo Fisher Scientific 14185052), the pieces were collected by centrifugation at 400g 5 minutes and digested using the NTDK Neural Tissue Dissociation Kit (P) (Miltenyi Biotec 130–092-628) at 37° C for 30 minutes following the manufacturer’s guidelines. Digested samples were quenched using ice-cold HBSS 1X and filtered through a 70 μm cell strainer, resuspended in 33% isotonic Percoll PLUS (GE Healthcare GE17–5445-01) and spun at 700 g for 15 minutes for myelin removal. The cell pellet was washed with FACS-BL and kept on ice for the following procedures.

To isolate macrophage from liver, heart and lung the organs were minced and digested in 2 mL of HBSS 1X buffer supplemented with 0.125 mg/mL Liberase TM (Sigma 05401119001), 0.5 mg/mL Collagenase type II (Sigma C6885) and 0.3 mg/mL Dnase I (Worthington Biochemical Corporation LS002007) at 37°C for 30 minutes, then filtered through 100 μm cell strainer (liver and lung) and 40 μm cell strainer (heart), resuspended in 33% isotonic isotonic Percoll PLUS (GE Healthcare GE17–5445-01) and spun at 700g for 15 minutes for fat removal. The cell pellet was then washed with MACS buffer [PBS 1x, 0.5 % bovine serum albumin (Sigma 10735078001) and 0.002 mM EDTA pH 8.0 (Thermo Fisher Scientific 15–575-020)], spun at 400 g for 5 minutes and kept on ice for the following procedures.

### Histological analyses

Brain, liver, and eye were collected from mice following transcardial perfusion with cold PBS-1X followed by overnight fixation in 4% paraformaldehyde (PFA) in PBS-1X. Fixed samples were washed once with PBS-1X and transferred to a 30% sucrose solution overnight for cryoprotection, embedded in Tissue-Tek optimal cutting temperature (OCT) compound, and cut (20 μm sections) on a cryostat (Leica, Wetzlar, Germany, CM3050). Tissues and serial sections were stored at −20°C until further use. To image transplant-derived GFP+ cells in the brain, spinal cord, retina, and liver the slides were then washed once in PBS-1X++, counterstained with Hoechst 3342 diluted 1 to 1000 in PBS-1X (PI62249, Thermo Fisher Scientific), and mounted in Aqua Poly/Mount (Polysciences) for fluorescent microscopy. The number of GFP+ cells in the retina was counted using 20X composite images of a whole retinal section (nasal-temporal). The count was performed by a researcher blinded to the treatment cohorts.

For immunofluorescence staining, slides were washed in PBS-1X + 1 mM CaCl2 + 0.5mM MgCl2 (abbreviated as PBS-1X++) to remove excess OCT. Sections were blocked and permeabilized in PBS-1X++ containing 0.2 % Triton X-100 and 10% normal goat or normal donkey serum (NGS or NDS, respectively; Gibco) for 1 h at 25°C. Primary antibodies were incubated overnight in PBS-1X++ with 5% NGS/NSDS at 4°C. Secondary antibodies were incubated in PBS-1X++ with 5% NGS/NDS 90 minutes at 25°C. Slides were then washed in PBS-1X++, counterstained with Hoechst 3342 diluted 1 to 1000 in PBS-1X (PI62249, Thermo Fisher Scientific), and mounted in Aqua Poly/Mount (Polysciences) for fluorescent microscopy. To stain microglia and myeloid cells, we used a rabbit anti-mouse Iba-1 antibody diluted 1 to 100 (AB 8395040, FUJIFILM Wako Pure Chemical Corporation) and a secondary Goat anti-rabbit Alexa Fluor 555 antibody diluted 1:500 (Thermo Fisher Scientific). To stain liver macrophages, we used a rat anti-mouse F4/80 antibody diluted 1 to 100 (clone BM8.1, Cell Signaling Technology) and a secondary Donkey anti-rat Cy3 antibody diluted 1:500 (Fisher Scientific). Slides were visualized by conventional epifluorescence microscopy using an all-in-one Fluorescence Microscope BZ-X800 (Keyence, Itasca, IL, USA).

### Cytokine measurement in mouse tissues and plasma

Ten/thirteen-week-old C57BL/6 mice were conditioned with Busulfan (100 mg/kg, n = 12; 25 mg/kg/day, from day −1 pre-transplant to day 4 post-transplant) or left untreated (Untreated, n = 3). Fifteen days after bone marrow transplant the Busulfan-conditioned mice (n =12) received PLX3397 (Pexidartib, MedChemExpress HY-16749) via oral gavage for six days (100 mg/kg/day). Mice were euthanized at 1, 4, 8 and 21 days after PLX3397 conditioning (n = 3 mice/day). Upon anesthesia (Ketamine 80 mg/kg, Xylazine 16 mg/kg) blood was collected in Safe-T-fill Dipotassium EDTA tubes (RAM Scientific, Nashville, TN, USA), and plasma was separated by centrifugation at 200 × g for 10 min. After blood collection, the mice were transcardially perfused with 1X-PBS. Bone marrow cells (BM) were collected by flushing femurs and tibiae using PBS-1X. Brains were dissected and one sagittal half/brain was snap frozen for cytokine analyses, while the other half was used for flow cytometry analyses. Brain and BM homogenates were prepared in RIPA Lysis buffer 1X (G Biosciences, St. Louis, MO, USA) supplemented with a protease inhibitor cocktail (Complete, Sigma-Aldrich). Brain and BM protein concentration was normalized using the BCA protein assay kit (Thermo Fisher Scientific). Mouse cytokines were quantified using the Immune Monitoring 48-Plex Mouse ProcartaPlex Panel (EPX480–20834-901, Thermo Fisher Scientific). Luminex assay was performed at the Human Immune Monitoring Center (HIMC, Stanford University School of Medicine, Stanford, CA, USA). Plasma samples were diluted 3 folds with PBS-1X and run in duplicates. Brain and BM homogenates, normalized to 5 mg/mL and 3 mg/mL, respectively, were run undiluted, in duplicates. ELISA assay was used to measure IL34 (DY5195–05, Mouse IL-34 DuoSet ELISA, R&D Systems) and SDF-1 (DY460, Mouse CXCL12/SDF-1 DuoSet ELISA, R&D Systems) cytokines in brain homogenates following the manufacturer’s instructions.

### Behavior study in wild-type mice and Grn−/− mice

Mice were group housed under reversed light cycle (8:30 am Light OFF-8:30 pm Light ON) and behavior tested during the animal dark cycle by Stanford’s Behavioral and Functional Neuroscience Laboratory (SBFN) by an experimenter who was blinded to the genotypes. In one study eight-week-old C57BL/6J mice (Jax strain #000664) were either untreated (n=10, 5 males and 5 females), conditioned by Busulfan (n=10, 5 males and 5 females) or Busulfan with PLX3397 and transplanted with total bone marrow as detailed above. Behavioral analyses were performed between 17 and 19 weeks after bone marrow transplant, corresponding to 14–16 weeks following the end of the PLX3397 administration, at these time points the mice were 7-month-old. In another study eight-week-old male B6(Cg)-Grntm1.1Aidi/J (Jax Strain #013175, abbreviated as Grn−/− or GRN KO, n=12) and age-matched male C57BL/6J mice (Jax strain #000664, n=15) underwent behavioral analyses from 2 up to 12 months of age.

#### Activity Chamber.

The locomotor assessment took place in an Open Field Activity Arena (Med Associates Inc., St. Albans, VT. Model ENV-515) mounted with three planes of infrared detectors, within a specially designed sound attenuating chamber (Med Associates Inc., St. Albans, VT. MED-017M-027). The arena was 43cm (L) × 43cm (W) × 30cm (H), and the sound attenuating chamber was 74cm (L) × 60cm (W) × 60cm (H). The mice were placed in the corner of the testing arena and allowed to explore the arena for 10 minutes while being tracked by an automated tracking system. Parameters including distance moved, velocity, rearing, and times spent in periphery and center of the arena were analyzed. The periphery was defined as the zone 5 cm away from the arena wall. The arena was cleaned with 1% Virkon solution at the end of each trial.

#### Y-maze: Spontaneous Alternation.

The Y-Maze was made of plastic with 3 arms in a “Y” shape. The arms were labelled as Arm A, B, and C. Arm A was 20.32cm (L) × 5cm (W) 12.7cm (H), and Arm B and C were 15.24cm (L) × 5cm (W), and 12.7cm (H). The test is based on the willingness of rodents to explore a novel environment and designed to measure spontaneous alternations. A normal rodent would prefer to explore a different arm of the maze than the arm they previously visited. Animals were placed in the center of the maze facing the intersection between arms B and C. The first entry was excluded from data analysis due to potential influence from the experimenter. Using an overhead camera, the numbers of arm entries and alternations were recorded for 5 minutes. An arm entry was defined as when all four paws entered into a new arm of the maze. The apparatus was cleaned with 1% Virkon after each trial. Parameters including percent alternation rate and total number of entries into arms were analyzed.

#### Novel Place Recognition (NPR) and Novel-Object Recognition (NOR).

The Novel Place Recognition (NPR) task assesses the ability of a subject to detect that a familiar object has been moved to a new location. The Novel Object Recognition (NOR) task assesses the ability of a subject to detect that an object has been replaced with a different object. NPR/NOR testing was conducted in a plastic arena 52cm (L) × 52cm (W) × 40cm (H). The walls of the plastic arena were black, and the floor was white. A white index card 12.7cm (L) × 7.62cm (W) was affixed to one wall as a visual cue. Two objects were used for this test: the green tower (18cm H × 4cm L × 4cm W) and the white bottle (16cm H × 4cm L × 4cm W). The green tower was made of dark green plastic and had a square base. The white bottle was made of white plastic and had a circular base. Assignment of one object as the NPR object and the other as the novel object introduced during NOR was pseudorandomized to ensure results were not influenced by an innate preference for either object. The object configuration was also pseudorandomized across subjects. The NPR/NOR tests were conducted over 3 days. On the first day, habituation, mice were each placed in the center of the empty arena and allowed to explore freely for the duration of a 10-minute trial. The NPR assessment was conducted on the second day. For NPR training, the arena had three identical objects placed in three different corners 10 cm from the wall. The mice were placed in the center of the arena and allowed to explore for 10 minutes. At the end of the NPR training, mice were returned to their home cage for 3–4 minutes prior to NPR testing. For NPR testing, one object was moved to the previously empty corner. Mice were then placed in the center of the arena and allowed to explore for 5 minutes. NOR assessment was conducted on the third day. One of the objects was removed and replaced with a different object. The mice were again placed in the center of the arena for a 5-minute trial. The trials were recorded, and mice were tracked with the automated tracking system Ethovision XT (Noldus Information Technology, Wageningen, Netherlands). An interaction was defined as when the nose of the mouse was within 2cm an object. Arena and objects were cleaned with 1% Virkon between each trial.

#### 3-Chambers Social Test.

The 3-Chambers apparatus was made of clear Plexiglas 60 cm (L) × 40.5 cm (W) × 22 cm (H) equally divided into three chambers by two pieces of clear dividers with a small hole 10 cm (L) × 5 cm (H) for mice to transition into all 3 chambers. The experiment consisted of three 10 minutes trials: Habituation, Sociability, and Social Discrimination. During the Habituation, the subject mouse was placed in the middle chamber with the wire cups placed inside the left and right chambers. The subject mouse was allowed to explore all three chambers for 10 min. After 10 min the subject mouse was guided into the middle chamber without access to the left and right chamber. During Sociability trial, Stranger 1 (3–5 weeks old, same sex, novel juvenile mouse) was placed under the cup in the left chamber and a Novel Object (plastic cap) in the right chamber. The subject mouse was allowed to explore the three chambers for another 10 minutes. After Sociability test the subject mouse was placed back in the middle chamber. Social Discrimination begins by placing Stranger 2 under the cup in the left chamber and the Stranger 1 in the right chamber. The subject mouse is allowed to explore all three chambers again for another 10 minutes. The position of Stranger 1 and Stranger 2 in left and right chamber are pseudo randomized. The Stranger mice were only exposed to one subject mouse per day. The Stranger mice were habituated to the cups for 10 min × 3 days prior to testing to reduce stress and mobility under the cup. Each Stranger mouse was dedicated to single cup during the test and the cups were washed with 70% ethanol after the test. The subject mouse was monitored by Ethovision tracking system and the total time in chambers and interactions with Stranger mice/Novel Object were analyzed. Interaction was defined as sniffing or direct contact with the Stranger mice/Novel Object within 2 cm of the wire cup. Chambers Social Test was conducted in Grn−/− mice and wild type controls at 2, 4, 6, 9, and 12 months of age. New Stranger mice were used at each timepoint.

#### Passive Avoidance.

The Passive Avoidance Test (PAT) was conducted using GIMINI avoidance system (San Diego Instruments, San Diego, CA). This automated system contained two compartments which were separated by a guillotine door (gate). Both compartments had grid floors which delivered electric shocks, but one compartment was lit and another one was dark. The experiment consisted of 1 day of habituation, 1 day of training, and 1 day of testing. On habituation day, the mouse was placed in the lit compartment. After 30 sec acclimation, the gate was opened and the mouse was allowed to explore both compartments freely. The gate was programmed to close when the mouse entered the dark compartment preventing the mouse from returning to the lit compartment. The mouse was removed from the system 30 sec after entering the dark compartment. On the following day, Training Day, the mouse was placed in the lit compartment. After 30 sec of acclimation the gate was opened and the mouse was allowed to explore both compartments freely. The gate was closed after it entered the dark compartment and an electric shock (0.5 mA for 2 sec) was delivered 3 sec after the door was closed. The mouse remained in the dark compartment for additional 30 sec before being removed and returned to the home cage. On the following day, Day 1 Testing Day, the mouse was placed in the lit compartment. After 5 sec acclimation, the gate was opened. When the mouse entered the dark compartment, the gate was closed and trial ended. The mouse was returned to the home cage. Maximum duration of each trial was 300 sec after the door opened. The time between the gate opening and the mouse passing through the gate was recorded as latency time. The compartments were cleaned with 1% Virkon between each animal.

#### Grooming Behavior.

Mice were singly housed in new home cages for 20 minutes, video recorded. The videos were hand scored by the experimenter. The grooming behavior was defined as face-wiping, scratching/rubbing of head and ears, and full-body grooming including licking or brushing any part of the body.

#### Nesting Behavior.

The Nesting behavior was conducted in a new mouse home cage with clean bedding (Innovive cage (37.3 cm(L) × 23.4 cm(W) × 14.0 cm(H)) made of clear plastic. Mice were singly housed during the experiment and a 3 g cotton nestlet (Ancare Corp.) approximately 5 cm (L) × 5 cm (W) × 0.5 cm (H) was introduced into the cage during 1st hour of the dark cycle. A nestlet score of 1–5 was recorded at 7 hours and 24 hours after introduction of the nestlet. Nesting Score: score of 1 = More than 90% of the nestlet untouched; score of 2= 50–90% of the nestlet intact with nestlet partially shredded; score of 3= Nestlet shredded 50–90% but no identifiable nest site. Less than 50% of the nestlet remains intact, but less than 90% is within a quarter of the cage floor area. Nestlet is not gathered into a nest but is spread around the cage. The material may sometimes be in a broadly defined nest area; score of 4= More than 90% of the nestlet is torn and the nestlet is gathered into an identifiable nest within a quarter of the cage floor area. However, the nest has flat wall which is defined as less than 50% of the circumference of mouse body height when curled up on its side. score of 5= More than 90% of the nestlet is torn and the nest is a crater, with walls higher than 50% of the circumference of the mouse body height. If the nesting score is ambiguous (e. g. a nest with a score of 5, but more than 10% of the nestlet un-shredded) a score of 4.5 was assigned.

### Single-cell RNA sequencing (scRNA-seq)

Brain and bone marrow cells were isolated, stained, and FAC-sorted as described above. 10,000 cells from sorted population were processed by MedGenome Inc. (Foster City, CA, USA) using the Chromium Controller and the Chromium Next GEM Single Cell 3’ Reagent Kits (10x Genomics). The libraries were sequenced via Illumina Novaseq 6000 sequencer (Illumina, San Diego, CA). The Illumina raw BCL sequencing files were processed through the CellRanger software (10x Genomics) for generating FASTQ files and count matrixes (https://support.10xgenomics.com/single-cell-gene-expression/software/overview/welcome). Feature-barcode matrices obtained from cellranger count for all the samples were processed using the ‘Read10X()’ function from Seurat package (v4.0)^109^. Next, cell filtering was performed based on nfeatures (> 200 & <2500) and the percentage of counts from mitochondrial genes (percent mt <5) ^110^. Normalization and scaling were performed and the top 2000 genes with the highest standardized variance were used to identify significant principal components (PCs). Clustering was performed using a graph-based method with the FindClusters() function and the resolution parameter set to 0.2. The resulting clusters were visualized using Uniform Manifold Approximation and Projection for Dimension Reduction (UMAP; **Extended data Fig. 6a**). FindAllMarkers Seurat function was utilized to find markers for each cluster. Heatmaps showing the top 5 DEGs in each cluster (**Extended data Fig. 6b-e**) were generated using the DoHeatmap function of SEURAT v4.0^109^. Cell annotation was generated by using the ScSorter R package with a pre-curated list of markers^111–113^. With the annotations from ScSorter, we identified markers in each population and and the top 5 DEGs in each population were visualized on a heatmap (**Extended data Fig.6f-g**).

To make comparisons between the four sorted cell populations we combined the count matrices in a single and used it as input for Scanpy (V1.9.3) Python tool for single cell analysis. Single cell barcodes were filtered for cells containing less than 5% mitochondrial gene counts^110^. Cells containing less than 200 genes, cells with very high gene counts (indicating doublets), and genes detected in less than three cells were excluded. Expression data were then normalized, log-transformed, and searched for variable features using the highly_variable_genes function followed by PCA and UMAP dimensionality reduction. Leiden clustering was conducted with 0.5 resolution^114^. The top 25 DEGs in each cluster were determined using a student’s t-test between each cluster and the combination of all remaining clusters.

### CyTOF staining run and analyses

Extraction of brain and bone marrow cells was performed as described above. Immediately after isolation the cells were washed one time in MACS buffer, spun at 400 g for 5 minutes, resuspended in 100 uL of MACS buffer and fixed using PROT1 (Smart Tube Inc proteomic Stablizer, Smart Tube) using the manufacturer’s instruction (MACS buffer: PROT1 ration 1:1.4)^115^. Upon 10 minutes incubation at 22°C the cells were frozen in dry ice and stored at −80°C. To process samples for CyTOF analysis, the samples were thawed 10 minutes at 4°C and washed twice with 1 mL of cell-staining medium (CSM; PBS with 0.5% BSA and 0.02% sodium azide, 2 mM EDTA pH 5) before proceeding with the staining using the surface and intracellular markers described in **Supplementary Table 3**. Following the two CSM washes the cells were resuspended in 10% vol/vol Mouse BD Fc Block^™^ (clone 2.4G2 BD Biosciences) in 50 μL of CSM and incubated 10 minutes on ice. Master mixes of antibodies for surface (50 μL/sample) and intracellular (100 μL/sample) markers were prepared in CSM separately and filtered using a 0.1 μM filter. The stainings were performed for 30 minutes at 22 °C on a shaker (300 rpm). After the surface staining the cells were washed with 4 mL of CSM and permeabilized with 1 mL of ice-cold methanol 100% for 15 minutes at 4°C. Samples were then washed twice with 4 mL of CSM and then stained with the antibody mix for the intracellular markers. Cells were then washed with 4 mL of CSM and stained with 1:5000 191Ir/193Ir DNA intercalator (Fluidigm) in 1.6% PFA in PBS-1X overnight at 4 °C. The following day the cells were washed with 4 mL CSM and 2 × 4 mL ddH2O, filtered and resuspended in 139La/142Pr/59Tb/160Tm/175Lu normalization beads before being analyzed using a Helios mass cytometer (Fluidigm) at 200 events/sec rate. IMD files were converted in FCS files and normalized with publicly available Matlab Normalizer v0.3 (https://github.com/nolanlab/bead-normalization)^116^. Raw count values were used to measure protein expression. FCS files were analyzed using OMIQ software from Dotmatics (https://www.omiq.ai/). Gated live cells (DNA+/cPARP−) were used for downstream analysis to calculate the percentage of positive cells or the expression of each marker. To identify cell subsets and to visualize single cell expression in 2D dimension, we run the optimized Stochastic Neighbor Embedding (Opt-SNE) on either live cells (DNA+/cPARP−) or CD45+ CD11b+ gated cells using all the markers for the clustering and the default settings for the run. Using the opt-SNE coordinates, we gated the GFP+ CD45+ CD11b+ cells (MGLCs) and the GFP− CD45+ CD11b+ cells. Surface and intracellular markers were gated on GFP+ MGLCs and the GFP− microglia (Fig. 4c, e, Fig. 5d and **Extended Data Fig. 8a**) or live cells (**Extended Data Fig. 8b**).

### Progranulin ELISA and Western blot

ELISA assay was used to measure GRN in serum of mice diluted 1 to 100–200 (DY2557–05, Mouse Progranulin DuoSet ELISA, R&D Systems), and brain and eye homogenates (AG-45A-0019YEK-KI01, Progranulin mouse ELISA Kit, AdipoGen) following the manufacturer’s instructions. Brain and eye homogenates were lysed in ELISA buffer [20 mM Tris,150 mM NaCl, 0.1% Triton supplemented with protease inhibitors (cOmplete^™^ Protease Inhibitor Cocktail, Roche)] using a Tissue Lyser II (20 Hz for 2 minutes, Qiagen). Protein quantification in the homogenates was performed by BCA (Pierce^™^ BCA Protein Assay Kits and Reagents, Thermo Scientific).

For Western blot analyses serum, brain, and eye lysates were denatured in Loading buffer-1X containing 1X Laemmli Sample Buffer (1610747, Thermo Scientific) and 1X Reducing Agent (novex NuPAGE Sample Reducing Agent, Invitrogen) at 95 °C for 5 minutes. Protein samples were loaded on 4–12% Bis-Tris Protein Gels (Nupage, Thermo Scientific) and run in MOPS SDS Running Buffer-1X (novex NuPAGE MOPS SDS Running Buffer, Invitrogen). Blotting was performed using iBlot2 Gel Transfer Device (Invitrogen). Membranes were blocked in Intercept TBS blocking buffer (LI-COR Biosciences). Primary antibodies were: sheep anti-mouse GRN (AF2557, R&D), rabbit anti-GFP (AB 221569, Thermo Scientific), mouse anti-alpha Tubulin (clone DM1A, T6199-Sigma-Aldrich). Total protein stain was performed used the Revert^™^ 700 Total Protein Stain Kits for Western Blot Normalization of serum samples (LI-COR Biosciences). Fluorophore-conjugated secondary antibodies used were: Donkey Anti-Rabbit IgG Polyclonal Antibody (IRDye^®^ 800CW, LI-COR Biosciences), Donkey Anti-Mouse IgG Polyclonal Antibody (IRDye^®^ 680RD, LI-COR Biosciences) and Donkey Anti-Sheep IgG Polyclonal Antibody (CF^™^ 680, Biotium). Membranes were scanned using the Odyssey XF imaging system (LI-COR Biosciences).

### Analysis of Bis(Monoacylglycero)Phosphate (BMP) in the brain.

Lipids were extracted as previously described^117^. Briefly, about 50 mg of brain tissue was homogenized in 150 μl KPBS with tissue disrupter and 25 μl of the homogenate were added to 1 ml lipid extraction buffer (Chloroform: Methanol, 2:1 v/v containing 750 ng ml^−1^ of SPLASH LIPIDOMIX internal standard mix from Avanti), followed by vortexing for 1 hour in a 4 °C cold room. Then, 200 μl of 0.9% (w/v) saline (VWR. Cat# L7528) were then added to the mixture and vortexed for 10 minutes in a 4 °C cold room. The mixture was then centrifuged at 3000 *×*g for 10 minutes at 4 °C to separate the methanol/saline (upper) and chloroform (lower) phases, the upper phase was discarded, and lipids-containing lower phase were collected to a new 1.5 ml tube and vacuum dried on SpeedVac. Lipid were reconstituted in 50 μl Acetonitrile:isopropanol:water 13:6:1 (v/v/v) for lipid quantitation as described below.

An Ascentis C18 column (5 Micron, 5 mum particle size, L × I.D. 5cm × 4,6mm) (Sigma-Aldrich) with an Ascentis Express guard holder that is connected to a 1290 LC system was used to separate lipids. The LC system was coupled to the 6470A triple quadrupole (QQQ) mass spectrometer to quantify targeted lipids as described^118^.

The mass spectrometer was operated in Multiple Reaction Method (MRM) for targeted analysis of species of interest. Standard BMP 18:1/18:1 (Avanti) was optimized using a MassHunter Optimizer software. The precursor-product ion pairs (m/z) used for MRM of the compounds were as follows: BMP 16:0/16:0 MNH_4_^+^, 740.54→313.27; BMP 16:0/18:1 MNH_4_^+^, 766.56→338.9/313.27; BMP 16:0/22:6 MNH_4_^+^, 812.54→385.27/313.27; BMP 16:1/18:1 MNH_4_^+^, 764.54→338.9/311.26; BMP 16:1/18:2 MNH_4_^+^, 762.54→336.9/311.26; BMP 16:1/20:3 MNH_4_^+^, 788.6→362.96/311.26; BMP 16:1/22:6 MNH_4_^+^, 810.53→385.27; BMP 18:0/18:1 MNH_4_^+^, 794.6→340.9/338.9; BMP 18:1/18:1 MNH_4_^+^, 792.6→338.9; BMP 18:1/18:2 MNH_4_^+^, 790.56→338.9/336.9; BMP 18:1/20:3 MNH_4_^+^, 816.56→363.27/338.9; BMP 18:1/20:4 MNH_4_^+^, 814.56→361.27/338.9; BMP 18:1/22:6 MNH_4_^+^, 838.56→385.27/338.9; BMP 18:2/20:3 MNH_4_^+^, 814.56→363.27/336.9; BMP 18:2/20:4 MNH_4_^+^, 812.54→361.27; BMP 18:2/22:6 MNH_4_^+^, 836.54→385.27/336.9; BMP 20:4/22:6 MNH_4_^+^, 860.54→385.27/361.27; BMP 22:5/22:6 MNH_4_^+^, 886.56→387.31/385.27; BMP 22:6/22:6 MNH_4_^+^, 884.54→385.27 and POPC: 760.6→124.9/60.2.

The annotation MNH4+ represents measurement in positive mode where M indicates the neutral mass while NH4+ is the ammonium adduct.

Lipids were annotated and quantified using a high-throughput analysis software called MassHunter for qualitative analysis and QQQ quantitative analysis software known as Quant-My-Way. The identification of BMP species in the figures was confirmed by manually verifying the peak alignment, matching retention times, and comparing MS/MS spectra to characteristic fragmentation patterns of standard compound. To ensure accurate identification and quantification, two transitions for each compound were analyzed, and their relative responses were compared. The MRM method and retention time were used for quantification of all lipid species with the quantification software. The raw abundances of all species were then exported to Microsoft Excel for further analysis. The abundances of BMP species were normalized to the abundance of endogenous control lipid in the same sample.

### Statistical analyses

All the data showed in the present manuscript are reported as Mean ± standard deviation of the mean. The number of sampled units, n, upon which we reported statistics is the single mouse for the in vivo experiments (one mouse is n = 1) or the number of independent experiments for in culture experiment (one experiment n=1). Mice that underwent neurobehavioral studies were serially evaluated overtime. GraphPad Prism 7 software (GraphPad Software) was used for statistical analyses. Parametric tests were used with data having a normal distribution (evaluated using the Shapiro–Wilk test). The statistical tests used were: unpaired Student’s t-test for two-group comparisons, One-way ANOVA with Tukey post hoc for comparisons with more than two groups, Two-way ANOVA with Tukey or Sidak post-hoc for multiple variable comparisons. For all the data sets analyzed by parametric tests, alpha=0.05. All statistical tests were performed two-sided. p-values <0.05 were considered significant. The statistical analysis performed for each data set is indicated in the figure legends. For all figures *p < 0.05, **p < 0.01, ***p < 0.001, ****p < 0.0001.

## Data Availability

Data supporting the findings of this work are available within the paper and its Supplementary Information files. A reporting summary for this Article is available as a Supplementary Information file. The datasets generated and analyzed during the current study are available from the corresponding authors upon request. Single-cell transcriptomic data generated in this publication will be deposited in NCBI’s Gene Expression Omnibus (GEO) database and will be available through GEO accession code.
